# The Nexus of Gut Microbiome, Microbial Metabolites, and Colonization Resistance Against Enteric Pathogens

**DOI:** 10.3390/biom16091291

**Published:** 2026-09-07

**Authors:** Mengwan Jiang, Mingke Yang, Peixuan Du, Zhongke Sun

**Affiliations:** 1 School of Artificial Intelligence and Big Data, Henan University of Technology, Zhengzhou 450001, China; 2 School of Biological Engineering, Henan University of Technology, Zhengzhou 450001, China

**Keywords:** gut microbial metabolites, pathogenic infection, colonization resistance, SCFAs, secondary bile acids, bacteriocins

## Abstract

The gut microbiome is a complex ecological system crucial to human physiology. Commensal microbes in the gut provide resistance against pathogenic colonization, mainly due to their metabolites. Though studies have revealed a few mechanisms of microbial metabolite-mediated colonization resistance, various commensal microbes in the gut produce versatile metabolites and confront different pathogens. To pave the way for microbial metabolite-based treatment, clarification of what microbes and derived metabolites contribute to colonization resistance is a central topic. This focused review addressed the nexus of the gut microbiome, microbial metabolites, and colonization resistance against three representative pathogens, namely *Clostridioides difficile*, *Salmonella enterica* subspecies *enterica serovar* Typhimurium, and vancomycin-resistant *Enterococcus*. Different microbes and microbial metabolites involved in colonization resistance against these pathogens are discussed. Microbial metabolites, mainly short-chain fatty acids, secondary bile acids, and bacteriocins, were included. The emerging role of signal molecules in combating pathogenic infections is also addressed. In contrast to others, we further discussed different strategies that can enhance microbial metabolite-mediated colonization resistance, such as precise microbial preparation, targeted proliferation of a protective metabolite-producing microbiome, regulation of dietary patterns to optimize metabolic homeostasis, enhancing the local concentration of metabolites in the gut, and host-matched intervention.

## 1. Introduction

A community of ~10^13^ microorganisms in the human gastrointestinal (GI) tract, termed the gut microbiome, is important for protection against pathogenic infections due to its protective, structural, and metabolic functions [1]. A few pathogenic infections can be effectively prevented or even cured by transplantation of the fecal or intestinal microbiome (FMT/IMT), such as infections caused by *Clostridioides difficile* (*C. difficile*). However, the success rates of FMT in these other settings have been variable, including common infections caused by *Salmonella enterica* subspecies *enterica serovar* Typhimurium (*S.* Typhimurium) and vancomycin-resistant *Enterococcus* (VRE) [2,3]. In fact, even in *C. difficile* infection (CDI), the success rate is variable, and additional environmental, ecological, or host factors influence FMT-based recovery [4]. Typical consumption of probiotic bacteria or a combination of probiotics shows limited efficacy in protecting against infections. It was proposed that a microbial consortium with complementary functions will be more effective and practicable instead [5].

Colonization resistance is believed to be the major mechanism employed by the host and the commensal microbiome to block pathogenic invasions through multilayered barriers [6]. As reported, the defined synthetic microbial consortia, including defined communities mimicking the normal gut microbiome, therapeutic communities, and minimal communities, have been investigated [7]. Microbial consortia with either determined or undetermined composition have been developed and tested for enhancing colonization resistance [8,9,10]. The consortium can be composed of strains belonging to either the same or different genera. For example, a community composed of three strains of lactobacilli significantly reduces *S*. Typhimurium adhesion and invasion in chickens [11]. A community of five lactobacilli strains and two *Bifidobacterium* strains significantly decreases *C. difficile* colonization in mice [12]. The composition of microbial consortia can be even more complex. A consortium named CBBP, consisting of *C. bolteae*, *Blautia producta*, *Bacteroides sartorii*, and *Parabacteroides distasonis*, restores colonization resistance and clears VRE from the intestines [13]. Similarly, a consortium composed of five strains belonging to five different genera efficiently restricts VRE gut colonization in antibiotic-treated mice [14]. However, it is recognized that commensal consortium-mediated colonization resistance to enteric pathogens is context-dependent [15,16]. In addition, the microbiome may even enhance pathogen expansion under inflammation. For example, colonization of *Bacteroidia* and *Clostridia* drives respiration-dependent *S.* Typhimurium expansion during colitis due to microbial-derived aspartate [17]. Therefore, despite the promise of microbiome research for the treatment of infectious diseases, microbiome-directed therapies are still limited [18].

Advances in microbial endocrinology suggest that commensal microbes produce various metabolites that influence a human’s health status [19]. These metabolites have complicated effects on the host immune system, including direct antagonism and indirect triggering of host responses to invading pathogens [20,21]. It was shown that there are marked alterations in stool metabolites in CDI patients, mapping 14 significantly dysregulated pathways, including tryptophan metabolism, bile acid metabolism, short-chain fatty acid metabolism, and fatty acid oxidation [22]. In fact, microbial metabolites have emerged as key players in the interplay between diet, the gut microbiome, and host health. Several major classes, such as short-chain fatty acids (SCFAs), secondary bile acids (SBAs), and bacteriocins, are recognized to regulate inflammatory, immune, and metabolic responses within the host [23,24,25]. Given that many infectious diseases are associated with dysbiosis of the gut microbiome and consequent reductions in microbial metabolite production, the administration of these metabolites represents a direct, multi-targeted treatment [26,27]. It is now believed that microbiome-based metabolite treatment may be a novel therapy that holds promise for various diseases [28,29].

A recent study concluded that CDI is associated with a distinct fecal metabolic milieu that may promote inflammation, impair colonization resistance, and facilitate pathogen replication [22]. However, it remains unknown whether the change in microbial metabolites is a cause or a consequence in gastrointestinal disease, though the concept that the gut microbiome influences a broad variety of human diseases through production or modification of metabolites is widely recognized [28]. A study reported that colonization resistance to *S.* Typhimurium is provided by *Bactericides*-derived propionate, which inhibits growth by disrupting intracellular pH homeostasis [30]. This is a good example that shows how commensal microbiome-derived metabolites mediate colonization resistance. However, *S.* Typhimurium can overcome the inhibitory effects of propionate by using it as a carbon source for anaerobic respiration, which means pathogens mitigate colonization resistance by metabolizing microbiome-derived propionate [31]. Meanwhile, it is widely known that not only do *Bacteroides* produce propionate, but also plenty of other metabolites produced by various commensal microbes contribute to improved colonization resistance.

Therefore, it is indispensable to know the associations between the gut microbiome, microbial metabolites, and colonization resistance. To make the complicated interplay simple, this work addresses the question with three representative pathogens, namely *C. difficile*, *S.* Typhimurium, and VRE. The methods for literature collection and screening are described as follows: By searching the database of PubMed with keywords “*Clostridioides difficile* AND colonization resistance”, “*Salmonella Typhimurium* AND colonization resistance”, and “vancomycin-resistant *Enterococcus* and colonization resistance”, respectively, all publications were collected based on the title and abstract at first. Then, the literature was classified as review or research articles. Thirdly, those without relation to the determined gut microbiome and microbial metabolites were discarded after reading the abstract. Only some landmark reviews and closely related research articles that were close to the topic and preferably published within the last ten years were cited in this work. As such, we will mainly discuss the following: (1) the type of gut microbes and how they act against infections of these three pathogens; (2) which metabolites derive from what microbes and how they contribute to colonization resistance, mainly focusing on SCFAs, SBAs, and bacteriocins; (3) the emerging role of signal molecules (such as indole, autoinducer-2, and fengycin) in combating pathogenic infections; and (4) how colonization resistance mediated by microbial metabolites can be enhanced.

## 2. Microbial Colonization and Factors Affecting Colonization Resistance

**Figure 1:**
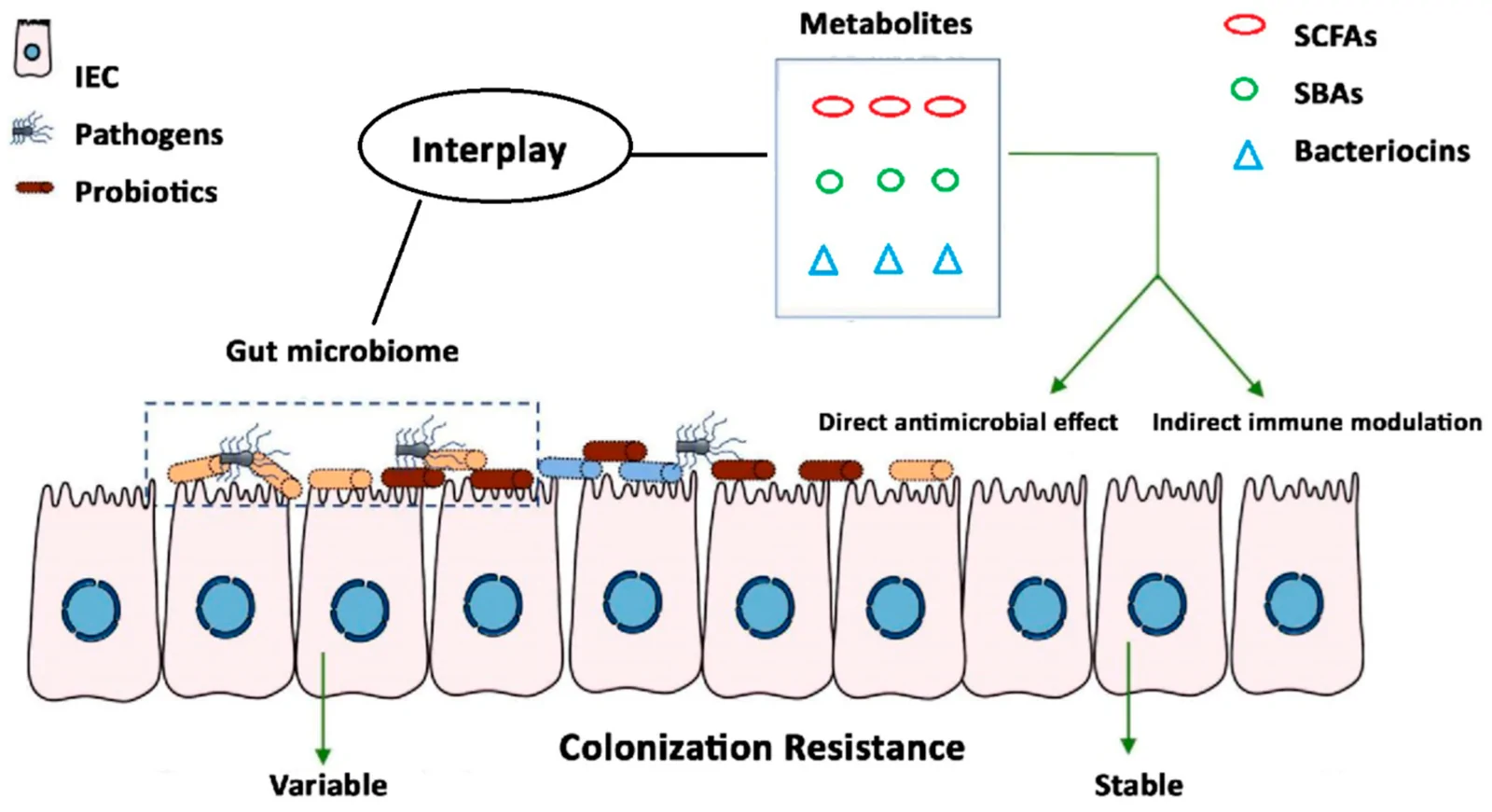
The interplay between the gut microbiome, microbial metabolites, and host determines colonization resistance against enteric pathogens. IEC, intestinal epithelium; SCFAs, short-chain fatty acids; SBAs, secondary bile acids.

Microbes can colonize a host, either on the surface of the skin or within tracts like the intestine. The commensal bacterium *Bacteroides thetaiotaomicron* (*B. thetaiotaomicron*) relies on the (p)ppGpp pathway by using alpha-ketoglutarate as a metabolic regulator, thereby halting its growth to adapt to carbon starvation and persist in the gut [32]. To colonize a host and expand infection, invading pathogens normally need to compete with the resident microbiome for niches and nutrients [33]. Current antibiotic treatments have led to increased susceptibility to infections and reduced colonization resistance. Re-establishing colonization resistance could markedly reduce infections, providing an alternative to unsustainable antibiotic therapy [34,35]. The commensal microbiome effectively inhibits colonization and overgrowth of pathogens in healthy humans. The general interrelationships between microbiome, microbial metabolites, and their impact on human health have already been discussed elsewhere [36]. As depicted in Figure 1, colonization resistance against enteric pathogens is determined by the gut microbiome, microbial metabolites, and host status. From the microbial perspective, factors related to microbial nutrient utilization, occupation of adhesion sites, production of metabolites, and modulation of host immune defense all influence colonization resistance [37]. Microbes directly suppress pathogens through niche and nutrient competition, contact-dependent inhibition, and the production of antimicrobial compounds and metabolites. From the host perspective, gut barriers and innate and adaptive immunity maintain microbiome homeostasis while selectively restricting pathogen expansion [38].

## 3. Microbes Involved in Gut Colonization Resistance

Gut microbes have complicated interactions with hosts. Many commensal microbes protect hosts against pathogenic infections by enhancing colonization resistance through direct or indirect interplay [39]. More than 10 years ago, a landmark review summarized the commensal microbes and how they confer colonization resistance to pathogenic infection from immunological studies [40]. The phyla of *Bacteroidetes*, *Firmicutes*, and *Actinobacteria* constitute the major intestinal microbiome combating pathogenic infections. Due to the large number of reported strains and rapid advancement, we here only list some of the well-investigated examples to illustrate what exactly these microbes are and how they enhance colonization resistance against three representative health-threatening pathogens based on literature published mainly between 2016–2026.

### 3.1. Microbes Against C. difficile Colonization

As an anaerobic toxigenic bacterium, *C. difficile* causes a severe infectious colitis that leads to significant morbidity and mortality worldwide [41]. Effective management of recurrent CDI remains a big challenge [42]. The strong protective effect of *Lachnospiraceae* was confirmed in Schaedler flora (ASF) mice, albeit the bacterium UBA3401 is currently unculturable [43]. UBA3401 is highly recalcitrant to growing without the presence of other commensal members. Its genome is similar to other Lachnospiraceae but contains a region closely homologous to a known thiopeptide bacteriocin gene cluster, including a YcaO enzyme at the end and multiple genes implicated in the regulation of biosynthesis, such as cyclic lactone autoinducers, as well as quorum sensing ranthipeptide genes. It was shown that probiotic *L. reuteri* alone does not offer a protective effect against *C. difficile*. However, *L. reuteri* can convert glycerol to the broad-spectrum antimicrobial compound reuterin. Therefore, co-delivery of *L. reuteri* with glycerol effectively protects against *C. difficile* colonization [44]. Significant decreases in *C. difficile* colonization by a probiotic consortium, including five *Lactobacilli* strains and two *Bifidobacterium* strains, have been reported in mice [12]. In contrast to those traditional probiotics, it has been demonstrated that *B. thetaiotaomicron* attenuates colonization of *C. difficile* and affects the intestinal microbiome [45,46]. Other inhabitants, like *C. scindens*, *C. butyricum*, and low-virulent *C. difficile*, are frequently found in the hospital environment and colonize hospitalized patients, suggesting they might be efficient competitors that occupy the living space and antagonize colonization of invading strains [47,48,49]. FMT suggests that increased colonization resistance to *C. difficile* is associated with bacteria classified within the *Lachnospiraceae*, *Ruminococcaceae*, as well as unclassified *Clostridiales* families [50]. A metagenomic study further suggests that key butyrate-producing bacteria, including *Christensenellaceae*, *Ruminococcaceae*, *Coprococcus*, *Fusicatenibacter*, *Oscillospira*, and *Roseburia*, might contribute to gut colonization resistance [51]. A more recent study concluded that the most significantly depleted taxa in the gut microbiome of patients with CDI versus that of the healthy control group were *Faecalibacterium prausnitzii* [52].

Except for bacteria, it was demonstrated that colonization with the human commensal fungus *Candida albicans* protects against lethal *C. difficile* infection in a murine model [53]. Probiotic yeast *Saccharomyces boulardii* CNCM I-745 inhibits *C. difficile* biofilm formation in vitro by modifying the extracellular matrix composition [54]. A retrospective matched-cohort study from 2016 to 2019 revealed that administration of *Saccharomyces boulardii* results in a significant risk reduction (48%) for inpatients receiving antibiotics associated with *C. difficile* infection [55]. In addition, several live biotherapeutic products comprising different undisclosed commensal strains have been developed for modulation of colonization, such as SER-109, VE303, RBX2660, and SMC B10 [8,9,10,56]. Taken together, different commensal microbes might be exploited to improve colonization resistance to *C. difficile*.

### 3.2. Microbes Against S. Typhimurium Colonization

*S.* Typhimurium is an important human pathogen responsible for millions of infections worldwide. It robustly colonizes the distal parts of the intestine such as the cecum and colon. It was found that the human commensal *E. faecium* is sufficient to protect mice against *S.* Typhimurium by promoting tolerance to the pathogen [57,58]. *Escherichia coli* Nissle 1917 limits the growth of *S.* Typhimurium and reduces its colonization in an inflamed intestine by competing for iron and secreted microcins [59,60]. It was further proposed that the commensal *Escherichia coli* Mt1B1 provides resistance by depleting galactitol, a carbon source that fuels *S.* Typhimurium colonization in mice [61]. *L. rhamnosus* P118 enhanced host tolerance to *S.* Typhimurium infection by promoting microbe-derived indole [62]. *L. rhamnosus* CIQ249 enhances mucosal defense against *S.* Typhimurium by strengthening the epithelial barrier and activating coordinated dendritic cell activation, T follicular helper cell differentiation, and IgA secretion [63]. Three strains of lactobacilli, namely *L. bulgaricus*, *L. rhamnosus*, and *L. paracasei*, significantly reduce *S.* Typhimurium adhesion and invasion in chicken cecal epithelial cells and virulence gene expression [11]. *Bacteroides* spp. provide gut resistance by disrupting the intracellular pH homeostasis of *S*. Typhimurium, though such benefit can be abolished in the intestinal lumen of Salmonella-infected germ-free mice due to anaerobic respiration [30,31]. The strain *Mucispirillum schaedleri*, present in the mammalian microbiome, has also been linked to host protection by interfering with invasion gene expression and competing for anaerobic electron acceptors [64].

In addition to these identified microbes, a minimal bacterial community provides colonization resistance against *S.* Typhimurium as well. A community harboring three facultative anaerobic bacteria provided conventional-like colonization resistance to *S.* Typhimurium [65]. Moreover, a restricted community of cultivable intestinal commensals protects susceptible mice from *S*. Typhimurium colonization by enhancing antibacterial IFNγ expression [66]. A significant decrease in total counts of *Salmonella* has also been observed in the ceca of layer chickens treated with symbiont segmented filamentous bacteria, which increases immune maturation [67]. Therefore, it seems that both single isolates and a collection of certain genera are effective in combating *Salmonella* infection, though their mechanisms are quite different. The mechanisms of colonization resistance mediated by those microbes include nutrients, production of inhibitory substances, and priming protective immune responses.

### 3.3. Microbes Against VRE Colonization

Due to the increasing development of antibiotic resistance, increasing length of hospitalizations, and excess mortality, VRE is an urgent and serious threat in hospitals. It has been demonstrated that *L. paracasei* CNCM I-3689 significantly reduces VRE numbers in mouse feces, which is associated with a better recovery of members of the phylum *Bacteroidetes* [68]. Two lactobacilli strains (*L. murinus* Y74 and *L. plantarum* HT121) separately decrease the colonization of VRE in the mouse gut through restoring gut microbiome diversity and down-regulating host defense-related genes such as defensin α, Apoa1, and RegIII [69]. Although *L. rhamnosus* GG, one of the most widely used probiotic strains, may facilitate decolonization of VRE from the human gut, an inconsistent effect was observed [70]. The enhancement of colonization resistance against VRE by other microbes has also been reported. A single antibiotic-susceptible strain, *E. faecalis* X98, significantly reduced VRE burden both in mouse and in vitro experiments, whereas multi-strain consortia failed due to competitive interference among consortium members [71]. A four-strain consortium, named CBBP, consisting of *C. bolteae*, *Blautia producta*, *Bacteroides sartorii*, and *Parabacteroides distasonis*, reverses antibiotic-induced susceptibility to VRE infection [72]. In fact, the consortium not only restores colonization resistance but also clears VRE from the intestines of antibiotic-treated mice [13]. A consortium composed of five strains belonging to the genera of *Alistipes*, *Barnesiella*, *Olsenella*, *Oscillibacter*, *Flavonifractor*, and *Bacteroides* is sufficient to restrict VRE gut colonization in antibiotic-treated mice [14]. Moreover, the study isolated a single bacterium (*Olsenella* sp.) that could recapitulate the effect of the consortium. By combining biological data and mathematical modeling, 15 molecular species (OTUs) that negatively correlated with VRE overgrowth were identified [16]. Representatives of these OTUs were used in combination with a seventh strain (Mix7), of which three belonged to *Lachnospiraceae*, one to *Muribaculaceae*, one to *Ruminococcaceae*, and two to *Lactobacillaceae*. Mix7 led to a better recovery of the gut microbiome composition and reduced VRE carriage (Table 1). Clearly, these microbes confer colonization resistance to VRE either by inducing host immunity or by the production of specific metabolites that have adverse effects on VRE.

## 4. Microbial Metabolites Contributing to Colonization Resistance

After determination of the representative microbes, it is essential to know what metabolites derived from them contribute to colonization resistance. In the human gut, there are different substrates metabolized by both host and microbes. Many host-derived metabolites can further be converted into secondary metabolites by microbes. Some microbial metabolites contribute to colonization resistance, either by inhibiting the growth of pathogens or regulating host immunity [42]. Versatile substances, such as exopolysaccharide, succinate, ethanolamine, N-acetyltryptophan, biotin, and microbiome-accessible carbohydrates, have been linked to bacterial colonization. Generally, the most widely recognized gut microbial metabolites related to colonization can be classified into three categories, including carbohydrate-related metabolites, amino acid-related metabolites, and bile acid-related metabolites [17,20,73]. We here present some representative metabolites, e.g., SCFAs, bacteriocins, and secondary bile acids (SBAs), respectively, related to three kinds of pathogens (Table 2).

### 4.1. Short-Chain Fatty Acids

The SCFAs mainly include acetate, propionate, and butyrate, and have different roles in the intestine. Acetate and propionate are mainly produced by *Bacteroidetes*, while butyrate is mainly metabolized by *Firmicutes* [74]. To prevent *S.* Typhimurium infection, it was demonstrated that SCFAs bind to apoptosis-associated speck-like protein to activate the inflammasome complex [75]. Thus, pathogens may monitor environmental SCFAs before initiating production of the colonization factors necessary for commensalism and persistence in favored niches. For example, a conceptual model proposed that *C. difficile* senses and responds to SCFAs as a marker of a healthy gut and tunes its virulence accordingly to maintain dysbiosis [76]. Microbial SCFAs might also modulate colonization resistance through regulating the expression of genes that keep bacterial growth and colonization under control. The correlation between SCFA species and growth inhibition of VRE has also been demonstrated [77]. Moreover, it was found that propionate and butyrate, but not acetate, induce histone acetylation of basophils and enhance IgE-mediated basophil degranulation [78]. To provide precision control, we elaborate on them one by one in the following sections.

More than forty years ago, it was found that acidification with acetate reduces the viability of *S*. Typhimurium cells in a variety of humectant solutions. Later, acetate produced by bifidobacteria was linked to improved epithelial barrier function and was proposed to protect the host against lethal enteropathogenic infection. The bactericidal effect of plasma-activated acetic acid on *S.* Typhimurium in chicken has been demonstrated [79]. For *C. difficile*, potassium acetate blocks its toxin A-induced tubulin deacetylation and subsequent microtubule disassembly that triggers barrier dysfunction in the gut by directly inhibiting histone deacetylase 6 [80]. In an acute mouse model infected with *C. difficile*, acetate enhances innate immune responses through coordinated action on both neutrophils and type 3 innate lymphoid cells [81]. In addition, acetate activates three kinds of histidine kinases and up-regulates bacteriocin synthesis in lactobacilli, both in vitro and in vivo, thereby reducing the number of pathogens [82].

Propionate has a plethora of health benefits, including antimicrobial and immune-modulatory effects [83]. For example, propionate induces RegIII lectins that are antimicrobial peptides at the mucosal surface of the gut [84]. Propionate also induces the expression of intestinal heat shock protein 70, which is potentially involved in intestinal homeostasis [85]. For *S.* Typhimurium, propionate specifically mediates colonization resistance to *S.* Typhimurium in mice [30]. Meanwhile, propionate reduces the viability of Salmonella in macrophages via propionylation of PhoP [86]. For VRE, propionate has a bacteriostatic effect, inhibiting growth in a dose-dependent manner [87].

Similar to acetate and propionate, butyrate promotes the acylation of key bacterial proteins and prevents the invasion of pathogenic bacteria by regulating the barrier function and immune status of the host gut [88]. In fact, butyrate drives monocyte-to-macrophage differentiation through histone deacetylase 3 (HDAC3) inhibition and induces antimicrobial activity in intestinal macrophages, thereby increasing resistance to enteric pathogens [89]. Butyrate significantly decreases the development and severity of *C. difficile* infection-induced colitis in mice by regulating bile acid metabolism and farnesoid X activation [90]. Another study reveals that butyrate attenuates intestinal inflammation and improves intestinal barrier function in infected mice, as shown by reduced intestinal epithelial permeability and bacterial translocation [81]. A study showed growth inhibition by exogenous butyrate and highlighted butyrate as a signal to alter *C. difficile* virulence by changing toxin production and spore formation [91]. Similar to the previous study, decreased growth of *C. difficile* in a butyrate dose-dependent manner has been reported, and the presence of butyrate also increases *C. difficile* sporulation [92]. Also, it has been found that butyrate effectively inhibits *S.* Typhimurium biofilm formation and reduces bacterial motility, which are important properties for pathogen adhesion [93]. For VRE, it has been reported that butyrate in combination with *Enterococcus faecalis*-derived lipoteichoic acid markedly enhances caspase-1 activation and IL-1β secretion, thereby facilitating inflammasome activation in macrophages [94].

### 4.2. Secondary Bile Acids

Secondary bile acids (SBAs) are metabolites produced by the commensal microbiome from primary bile acids secreted in the liver. The gut microbiome transforms unabsorbed primary bile acids via deconjugation, dehydrogenation, and dehydroxylation, producing mainly deoxycholic acid (DCA) from cholic acid and lithocholic acid (LCA) from chenodeoxycholic acid in the colon [95]. SBAs have various direct interplays in the human body depending on the conformation, abundance, and location of specific SBAs [96]. Their roles in *C. difficile* pathogenesis have been reviewed in detail [97]. For example, many SBAs, including lithocholate and its epimers, can inhibit taurocholate-mediated *C. difficile* spore germination, growth, and toxin activity in a dose-dependent manner [98,99]. Administration of a 7α-dehydroxylating *C. scindens* enhances colonization resistance against *C. difficile* in an SBA-dependent fashion and is due to normalization of the composition of intestinal deoxycholate and lithocholate [100]. On one hand, SBAs repress invasion gene (e.g., *Salmonella* pathogenicity island-6) expression by directly binding and inhibiting the function of HilD, which is an important regulator of *S.* Typhimurium virulence and pathogenesis [101,102]. On the other hand, SBAs like chenodeoxycholic acid reduce *S.* Typhimurium by triggering an antimicrobial program via up-regulated expression of Paneth cell α-defensins and increased synthesis of RegIIIb and RegIIIg by the ileal epithelium, after oral administration [103]. However, it has been discovered that LCA impairs separation of growing VRE diplococci, causing the formation of long chains and increased biofilm formation [104].

### 4.3. Bacteriocins

Bacteriocins are a diverse group of antimicrobial peptides that are toxic to a narrow spectrum of bacteria. They are important weapons employed by the commensal microbiome for improving colonization resistance due to improved fitness and competitiveness [105]. Microcins, produced by *Eschericha coli* Nissle 1917, inhibit enteric pathogens and reduce enterobacterial blooms [59]. High-molecular-weight bacteriocins, such as R-type diffocins, act as molecular puncture devices that specifically penetrate the cell envelope of some *C. difficile* strains to dissipate the membrane potential and kill the attacked bacterium [106]. As a particularly effective anti-*C. difficile* agent, micrococcin P2 is even better than vancomycin and fidaxomicin in mice [107]. Using an ex vivo model of the human colon, it was demonstrated that nisin selectively depletes *C. difficile* (e.g., no viable cells can be detected in the presence of 50–500 µM nisin) in a fecal microbial environment [108]. It seems lantibiotics (including nisin, actagardine, mersacidin, NAI-107 and MU-1140) are promising candidates for the treatment of *C. difficile* infections due to a narrow sensitivity profile and minimal impact on the protective gut microbiome when compared to traditional agents [109]. *Blautia producta* BP_SCSK_ produced a lantibiotic that reduces colonization and restores resistance against VRE in mice [13]. It has been reported that several bacteriocins have activity against VRE, such as nisin, bacteriocin EF478, enterocin P, enterocin K1, and EJ97 [110]. For *S.* Typhimurium, nisin (when the concentration is above 1.2 mg/mL) is highly effective in reducing bacterial biofilm formation, and a synergistic effect has been observed when combined with EDTA [111]. A novel bacteriocin derivative (A11), modified from acidocin J1132β (produced by *L. acidophilus*), shows strong antimicrobial activity against *S.* Typhimurium. A11 causes transient membrane permeabilization and kills bacterial cells through membrane depolarization and/or intracellular interactions with bacterial DNA [112] (Table 2).

**Table 2:** Microbial metabolites confer colonization resistance to three enteric pathogens.

Class	Metabolite	Target Pathogen	Mechanism	Reference
SCFAs	Acetate	*C. difficile*	Blocks toxin A-induced tubulin deacetylation and subsequent microtubule disassembly; inhibits histone deacetylase 6	[80]
	Acetate	*C. difficile*	Enhances innate immune responses through coordinated action on both neutrophils and type 3 innate lymphoid cells	[81]
	Acetate	NA	Activates three kinds of histidine kinases and up-regulates bacteriocin synthesis in lactobacilli	[82]
	Propionate	*S. Typhimurium*	Inhibits pathogen growth by disrupting intracellular pH homeostasis	[30]
	Propionate	*S. Typhimurium*	Reduces the viability of Salmonella in macrophages via propionylation of PhoP	[86]
	Propionate	NA	Induces RegIII lectins that are antimicrobial peptides at the mucosal surface of the gut Induces expression of intestinal heat shock protein 70, which is potentially involved in intestinal homeostasis	[84,85]
	Propionate	VRE	Inhibits growth in a dose-dependent manner	[87]
	Butyrate	*C. difficile*	Regulates bile acid metabolism and farnesoid X activation	[90]
	Butyrate	*C. difficile*	Alters *C. difficile* virulence by changing toxin production and spore formation	[91]
	Butyrate	*C. difficile*	Decreased growth of *C. difficile* in a butyrate dose-dependent manner	[92]
	Butyrate	*S.* Typhimurium	Inhibits *S.* Typhimurium biofilm formation and reduces bacterial motility	[93]
	Butyrate	NA	Drives monocyte to macrophage differentiation and induces antimicrobial activity in intestinal macrophages	[89]
	Butyrate	NA	Reduces intestinal epithelial permeability and bacterial translocation	[81]
	Butyrate	VRE	Enhances caspase-1 activation and IL-1β secretion, thereby facilitating inflammasome activation in macrophages	[94]
SBAs	Lithocholate and its epimers	*C. difficile*	Inhibits taurocholate-mediated *C. difficile* spore germination, growth, and toxin activity in a dose-dependent manner	[98,99]
	Deoxycholate and lithocholate	*C. difficile*	7α-dehydroxylaed SBAs normalize the large intestinal bile acid composition	[100]
	Lithocholic acid	*S.* Typhimurium	Represses invasion gene (e.g., Salmonella pathogenicity island-6) expression by directly binding and inhibiting the function of HilD	[101,102]
	Chenodeoxycholic acid	*S.* Typhimurium	Triggers an antimicrobial program via up-regulated expression of Paneth cell α-defensins and increased synthesis of RegIIIb and RegIIIg by the ileal epithelium	[103]
Bacteroicins	R-type diffocins	*C. difficile*	Penetrates the cell envelope of some *C. difficile* strains to dissipate the membrane potential and kill the attacked bacterium	[106]
	Micrococcin P2	*C. difficile*	Anti-*C. difficile* agent, is even better than vancomycin and fidaxomicin in mice	[107]
	Nisin	*S.* Typhimurium	Reduces bacterial biofilm formation	[111]
	Microcins	*S.* Typhimurium	Microcins mediate inter- and intraspecies competition among Enterobacteriaceae; inhibit growth and reduce colonization	[59]
	Bacteriocin derivative A11 modified from acidocin J1132β	*S.* Typhimurium	Causes transient membrane permeabilization and kills bacterial cells through membrane depolarization and/or intracellular interactions with bacterial DNA	[112]
	Lantibiotics	VRE	Reduces colonization and restores resistance against VRE in mice	[13,110]

NA, not available; SCFAs, short-chain fatty acids; SBAs, secondary bile acids.

## 5. The Emerging Role of Signal Molecules in Colonization Resistance

Pathogens respond to and integrate a myriad of signal molecules to control virulence and colonization [113]. One signal is indole, which is produced by several bacteria in large quantities and is widespread in the intestinal tract. As a microbial metabolite of tryptophan, indole strengthens the mucosal barrier and mucin production, and inhibits *Salmonella* virulence [114,115]. Indole and its derivatives are potential anti-virulence compounds against antibiotic-resistant pathogens since they can inhibit quorum sensing and virulence factor production [116]. A compound, CZ74 (a derivative of indole), shows high antibacterial potency against VRE by disrupting FtsZ polymerization and inhibiting GTPase activity and cell division [117]. Increased colonization resistance to *S.* Typhimurium and enhanced inhibitory effects of SCFAs on SPI-1 gene expression in IECs have been linked to indole [114]. The protective effects of *L. rhamnosus* P118 against *S*. Typhimurium are largely due to increased fecal levels of tryptophan and its derivatives (indole, indole-3-acrylic acid, 5-hydroxytryptophan, and 5-methoxyindoleacetate) [62]. However, it was reported that high levels of indole might allow *C. difficile* to proliferate and persist in the colon by restricting the growth of beneficial indole-sensitive bacteria and altering colonization resistance [118].

Autoinducers (AIs), another kind of signal molecules, are small metabolites used by bacteria as a common language for inter-/intraspecies communication. AIs enable synchronized behavior in response to environmental conditions and control virulence gene expression in many pathogens. In *S.* Typhimurium, AI-2 induces c-di-GMP synthesis to repress type III secretion system (T3SS) effector gene expression [119]. In addition, AI-3 affects the expression of almost 8% of all genes in *S.* Typhimurium, and several of them are involved in gut colonization [120]. Other less-studied microbial signal molecules also contribute to colonization resistance [121]. In the ileum, formic acid (as a cytoplasmic signal) outcompetes other signals and triggers the activation of virulence genes in *S.* Typhimurium [122]. In addition, c-di-GMP signaling regulates *S.* Typhimurium biofilms, diffusible signal factors (DSFs, e.g., cis-2-unsaturated fatty acids), and represses the virulence gene expression of *S.* Typhimurium by interacting with transcriptional regulators and inhibiting Salmonella invasion [123,124].

## 6. Enhancement of Microbial Metabolites Mediated Colonization Resistance

**Figure 2:**
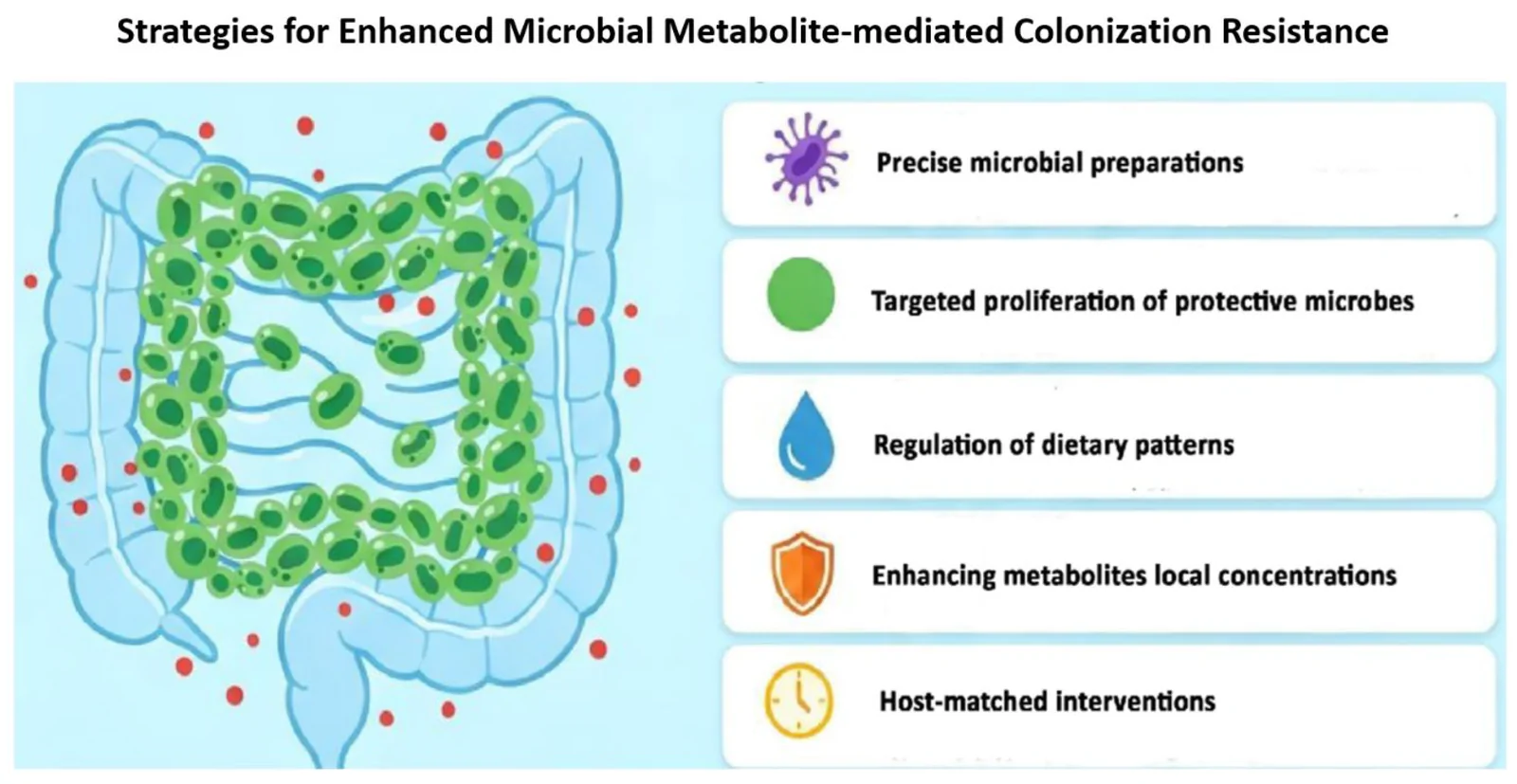
Strategies can be explored for enhanced microbial metabolite-mediated colonization resistance against three enteric pathogens.

Though colonization resistance is widely recognized, its successful establishment and maintenance through artificial interference with the gut microbiome and microbial metabolites is not easy. One reason for this is that the protective effects conferred by the microbiome and its metabolites are complex and include competitive microbial interactions and induction of host immune responses [125]. For example, various SBAs show diverse effects and modify virulence determinants, such as flagella expression, host cell adhesion, and toxin synthesis, differently in *C. difficile* [126]. Another reason is that pathogens have evolved multiple strategies to quickly subvert colonization resistance under dysbiosis. It was reported that *Enterococcus faecalis* adapts to decreased bile acids deoxycholate (DCA) and taurocholate (TCA) concentrations during dysbiosis and thereby expands its population, causing infection [127]. At present, the following strategies mainly include the following: (1) precise microbial preparation; (2) targeted proliferation of protective metabolite-producing microbiome; (3) regulation of dietary patterns to optimize metabolic homeostasis; (4) enhancement of the local concentration of metabolites in the gut; (5) and host-matched interventions, which are reported to enhance the efficiency of microbial metabolite-mediated colonization resistance (Figure 2).

### 6.1. Precise Microbial Preparation

For individuals with functional microorganism deficiency and insufficient metabolite synthesis (e.g., after antibiotic use, irritable bowel syndrome, recurrent intestinal infections), targeted supplementation of core functional bacterial strains and reconstructing metabolic pathways are mainstream strategies. These protective metabolite-producing bacteria are provided in the following four categories: (1) symbiotic bacteria, including *Bifidobacteria*, anaerobic bacillus, and complex Flavobacterium strains, directly colonize the colon and continuously secrete butyric acid, propionic acid, and succinate that significantly enhance resistance to pathogenic bacteria, especially *C. difficile* [51,73]; (2) bile acid conversion-specific strains, such as *C. scindens* single-strain/compound formulations, restore secondary bile acid metabolism, which requires an appropriate amount of dietary fiber as the carbon source for the strain [128]; (3) tryptophan metabolism probiotics, like *L. plantarum* and *Bifidobacterium longum*, enhance the production of indole derivatives, activate the IL-22 mucosal defense pathway, and resist invasion by pathogenic bacteria like *S.* Typhimurium [129]; (4) genetically engineered strains, such as *Escherichia coli* Nissle 1917, *L. casei*, and *E. faecalis*, express bacteriocins, enzymes, and immune-inducing proteins for improved tolerance to *S.* Typhimurium and VRE [130,131].

### 6.2. Targeted Proliferation of Protective Metabolite-Producing Microbiome

As a fundamental strategy, providing specific substrates for symbiotic bacteria that produce SCFAs, SBAs, and indole metabolites can continuously elevate protective metabolite levels and strengthen colonization resistance at the source. This can be achieved by an adequate intake of complex prebiotic dietary fibers to maximize SCFA production [132]. For example, oligosaccharides promote the growth of *Bifidobacterium* and lactobacilli species and generate acetate and lactate [133]. Distinct classes of prebiotics include oligosaccharides (fructo-oligosaccharides, arabinoxylan), nondigestible polysaccharides (inulin, pectin, resistant starch, golden kiwi fiber), and the vitamin riboflavin, which reduce the doubling time of *Faecalibacterium prausnitzii*, thereby boosting butyrate production [134,135]. Meanwhile, reducing refined sugars and high-fructose corn syrup, which decrease the abundance of *Ruminococcaceae* and *Lachnospiraceae* (representative butyrate-producing bacteria) and nourish pathogenic bacteria such as Enterobacteriaceae, should be avoided [136]. Targeted proliferation can also be manipulated by supplementing tryptophan and polyphenolic substrates that activate the indole-aryl hydrocarbon receptor pathway [137]. High-quality plant-based tryptophan sources, such as chickpeas, black beans, nuts, and tofu, provide precursors for indole metabolism. Polyphenols selectively promote *Akkermansia muciniphila*, which thickens the mucus layer and synergistically boosts indole propionate and other indole metabolites [138,139].

The combination of probiotics and specific matching prebiotics can avoid rapid death of the strains due to a lack of substrates after colonization. For example, the metabolic output is more than twice that of a single probiotic when lactobacilli are combined with galacto-oligosaccharides and butyric acid-producing bacteria are combined with resistant starch [140,141]. Consuming moderate amounts of ω-3 polyunsaturated fatty acids (deep-sea fish, flaxseeds, or chia seeds) promotes an abundance of butyrate-producing bacteria, including *Clostridiaceae*, *Sutterellaceae*, and *Akkermansiaceae* [142]. For example, the protective effect of dietary α-linolenic acid in chickens against *S.* Typhimurium infection is partially due to a remodeled gut microbiome [143]. Fermented foods provide live microbes and in situ metabolites. Sugar-free fermented products, such as yogurt, natto, and low-salt kimchi, supply lactobacilli and *Streptococcus* species, which colonize and continuously secrete lactic acid and bacteriocins. The breakdown of fermentation substrates generates small-molecule postbiotics, rapidly enhancing gut microbiome recovery and homeostasis [144].

### 6.3. Regulation of Dietary Patterns to Optimize Metabolic Homeostasis

Diet has a rapid and profound impact on gut microbiome composition and function and, consequently, colonization resistance. Time-restricted feeding or intermittent fasting is a well-recognized way to maintain health. A pilot study showed that an abundance of *Akkermansia muciniphila* and *Bacteroides fragilis* is significantly increased in all subjects after Islamic fasting [145]. In fact, fasting increases gut microbiome diversity and beneficial bacterial abundance, shifts microbial metabolism, and boosts SCFA and indole derivative production [146]. However, a randomized human intervention study concluded that caloric restriction disrupts the microbiome and colonization resistance [147]. Precisely, severe calorie restriction led to a decrease in bacterial abundance and a restructuring of the gut microbiome. Weight loss was associated with impaired nutrient absorption, enrichment in *C. difficile*, and a decrease in bile acids. As summarized, high-fat diets alter the microbiome and colonization resistance via bile acid production, while low-protein diets, in contrast, can enhance protection against *C. difficile*, possibly by reducing the free amino acids available to it [148].

It was demonstrated that whole dietary regimes, such as Mediterranean, high-fiber, and plant-based diets, have a profound impact on the stability, functionality, and diversity of the gut microbiome [149]. Better adherence to the Mediterranean diet was associated with significantly higher levels of total SCFAs, and volunteers who consumed plant-based nutrients, such as vegetable proteins and polysaccharides, showed higher bifidobacterial counts as well as higher total SCFAs [150]. Dietary protein, sugar, and amino acids may also be important regulators of colonization resistance. Even a short-term increase in specific ingested amino acids can reduce colonization resistance and enhance the growth of *S.* Typhimurium [151]. Consumption of a sucrose-rich diet reshaped the gut microbiome, marked by blooms of *Enterococcus* and *Akkermansia*, a reduction in beneficial taxa, and a remodeling of the metabolome to favor *C. difficile* germination and growth [152].

### 6.4. Enhancing the Local Concentration of Metabolites in the Gut

Postbiotics encompass diverse bioactive compounds such as SCFAs, exopolysaccharides, bacteriocins, antioxidant enzymes, surface layer proteins, and bacterial lysates, each exerting distinct biological effects [153]. Many metabolites, such as butyric acid glyceride, indole propionic acid, and purified bacteriocins, are suitable for individuals with severe microbiome imbalance, quickly repairing the inhibitory microenvironment, thereby restoring colonization resistance. However, regular nutrition or externally ingested metabolites are easily degraded in the stomach and small intestine and cannot reach target sites in the colon [154]. Colon-targeted delivery technology increases the local concentration [155,156]. Chitosan or pectin microspheres, which are resistant to stomach acid, encapsulate butyric acid, polyphenols, and probiotics, and only release after enzymatic degradation by colonic bacterial colonies, thereby increasing colonic SCFA concentration [157,158]. Tryptophan and probiotics encapsulated by modified resistant starch directly reached the distal colon and strengthened SBAs and indole metabolism [159,160]. In addition, direct supplementation of postbiotics can be quickly metabolized under emergency conditions [161].

Delivery by engineered symbiotic bacteria (modified by genetic/synthetic biology) is another promising way. Modifying probiotics to specifically secrete butyric acid and bacteriocins can continuously release defense metabolites and has already entered the clinical translation stage. For example, *Escherichia coli* Nissle 1917, capable of producing microcin M and microcin H47, can significantly inhibit *S.* Typhimurium both in vitro and in vivo [59]. This inhibition, however, was seen only during intestinal inflammation when *S.* Typhimurium expressed siderophores to scavenge iron from an iron-depleted environment. *Escherichia coli* Nissle 1917 has also been engineered to secrete three antimicrobial peptides, Enterocin A, Enterocin B, and Hiracin JM79, to specifically target and kill both *E. faecium* and *E. faecalis*, the two most prominent species responsible for VRE infection. Administration of the engineered probiotic significantly reduced levels of both pathogens in the feces of male mice [162]. Different from bacteriocins, the specific bacterial protein, the *Escherichia coli* Nissle 1917 outer membrane protein, MipA, induces the up-regulation of integrin-linked kinase, thereby reinforcing tight junction integrity. It was further shown that engineering a nonprotective strain to express MipA was sufficient to confer colonization resistance against Salmonella infection [163].

### 6.5. Host-Matched Intervention

Host-related interventions, such as regular exercise, adequate sleep, reduced alcohol consumption, and restricted use of disturbing drugs, can synergistically enhance the defensive effects of microbial metabolites. A meta-analysis concluded that voluntary running increased the thickness of duodenal villi and microbiome diversity, low-intensity treadmill running increased the abundance of Actinobacteria and Bifidobacteriaceae, while high-intensity swimming and treadmill running reduced SCFAs [164]. It was further reported that continuous moderate-intensity training (e.g., 30–60 min at 50–70% VO_2_ max) enhances tight junction proteins, reduces oxidative stress, and shifts cytokines toward anti-inflammatory profiles, therefore improving barrier integrity and enriching *Lactobacillus*, *Bifidobacterium*, *Faecalibacterium prausnitzii*, and *Roseburia hominis* [165]. The positive effect of moderate exercise on colonization resistance against methicillin-resistant *Staphylococcus aureus* (MRSA) was considered related to an understudied intestinal probiotic strain, *Dubosiella newyorkensis* L8 [166]. The strain L8 enhances the depletion of fucose, a crucial carbon source essential for MRSA growth and pathogenicity.

Sleep deprivation is another important contributor to the disturbance of intestinal homeostasis. It was shown that sleep deprivation impairs intestinal colonization resistance in mice, whereas nicotinamide mononucleotide supplementation restores it [167]. Specifically, the altered gut microbiome further incurs disorders of SBA pools, accompanied by a decrease in deoxycholic acid (DCA). In mice, acute sleep deprivation (ASD), induced by 72 h of rapid eye movement, led to disruption of the circadian rhythms, as well as gut microbiome dysbiosis [168]. These impacts were related to a decrement in gut microbiome diversity, SCFAs, gut inflammation, and hyperpermeability. For example, the relative abundance of *Lactobacillus*, *Muribaculum*, *Monoglobus*, and *Parasutterella* decreased in the ASD group. These effects were accompanied by a reduction in fecal propionic acid, higher inflammation, dysregulation of circadian rhythms, and tight junction genes. Analysis of a total of 20 studies showed that sleep deprivation significantly reduced alpha diversity (Shannon and Simpson indices) and increased the Firmicutes-to-Bacteroidetes ratio [169]. Reductions in *Lactobacillaceae* and *Erysipelotrichaceae* and increases in *Ruminococcaceae* and *Lachnospiraceae* were observed.

Several studies have shown that alcohol consumption is linked to the development of gut dysbiosis, largely due to the disruption of the tight junctions [170,171]. Using a mouse model, it was shown that chronic alcohol consumption alters the diversity and composition of the gut microbiome, disturbs gut metabolites, and promotes colonization of *Klebsiella pneumoniae* in the gut [172]. Further relevant analyses have found that gut microbiome dysbiosis is strongly correlated with alterations in SBAs; LCA may be a potential drug candidate for preventing colonization and the spread of pathogens. In fact, alcohol directly damages goblet cells, destroys the mucus barrier, and significantly weakens colonization resistance [173,174].

Antibiotics promote intestinal growth of resistant enteric pathogens by enriching nutrients and depleting microbial metabolites [175]. Broad-spectrum antibiotics indiscriminately kill symbiotic bacteria that convert butyric acid and bile acids, resulting in a significant decrease in SCFAs and SBAs and a complete collapse of colonization resistance [176]. For example, lantibiotic-mediated dysbiosis results in a sustained loss of colonization resistance against CDI [177]. Longer durations of piperacillin/tazobactam treatment caused prolonged microbiome disruption and alteration of colonization resistance against *C. difficile* and VRE [178]. Unlike traditional considerations, non-antibiotic drugs may also contribute to the development of enteric infections. It was demonstrated that 28% of the tested 53 d non-antibiotic drugs increased the growth of *S.* Typhimurium, indicating these drugs promoted pathogen proliferation and induced disruption of colonization resistance, consequently accelerating disease onset and increasing inflammation [179]. In contrast, natural product chlorotonil A (ChA) preserved the murine and porcine microbiome composition and minimally impacted the intestinal metabolome, and therefore does not interfere with colonization resistance against *C. difficile* [180]. Additionally, ChA accumulates in the spore and inhibits outgrowth of *C. difficile*, contributing to a lower CDI rate. Therefore, careful use of drugs may avoid damage to colonization resistance.

## 7. Conclusions

Although the transfer of a specific commensal community generally enhances colonization resistance, the effect is context-dependent and varies with different pathogens. In contrast, all these commensal microbial-derived metabolites, including SCFAs, SBAs, bacteriocins, signal molecules, and other undetermined substances, may contribute to improved colonization resistance in a more stable and ubiquitous manner. The efficiency of microbial metabolite-mediated colonization resistance may be improved by precise microbial preparation, targeted proliferation of the protective metabolite-producing microbiome, regulation of dietary patterns to optimize metabolic homeostasis, enhancement of the local concentration of metabolites in the gut, and host-matched interventions.

## Figures and Tables

**Table 1:** Representative microbes confer colonization resistance to three enteric pathogens.

Pathogen	Microbes	Models	Mechanisms	References
*C. difficile* *	*L. reuteri* plus glycerol	Mice	*L. reuteri* bacteria convert glycerol to the broad-spectrum antimicrobial compound reuterin	[44]
	*Bacteroides thetaiotaomicron*	Mice	Affects intestinal microbiota and bile acid profile and suppresses toxin release	[45]
	*Candida albicans*	Mice	Alters the gut ecosystem and enhances protective immunity	[53]
	*Saccharomyces boulardii* CNCM I-745	In vitro	Inhibits *C. difficile* biofilm formation by modifying the extracellular matrix composition	[54]
	A 4-bacteria consortium including *C. scindens* or *C. scindens* alone	Mice	Enhances resistance to infection in an SBA-dependent fashion due to its dehydroxylating ability of bile acids	[47]
	Five *Lactobacilli* strains and two *Bifidobacterium* strains	Mice	Alters gut microbiota and bile acids	[69]
*S.* Typhimurium	Commensal *E. faecium*	Caenorhabditis elegans and mice	A secreted bacterial peptidoglycan hydrolase; SagA generates muramyl-peptide fragments against *Salmonella* colonization	[57]
	*Escherichia coli* Nissle 1917	Mice	Produces microcin, limits growth, and competes for iron	[59,60]
	*Escherichia coli* Mt1B1	Mice	Depletes galactitol, a carbon source and substrate that fuels *S.* Typhimurium colonization	[61]
	*L. rhamnosus* P118	Mice	Promotes microbe-derived indole	[62]
	*L. rhamnosus* CIQ249	Mice	Strengthens the epithelial barrier and activates coordinated dendritic cell activation, T follicular helper cell differentiation, and IgA secretion	[63]
	*Bacteroides* spp.	Mice	*Bacteroides*-derived propionate directly inhibits pathogen growth by disrupting intracellular pH homeostasis	[30]
	*L. bulgaricus*, *L. rhamnosus*, and *L. paracasei*	chicken cecal epithelial cells	Reduces adhesion, invasion, and virulence gene expression	[11]
VRE	*L. paracasei* CNCM I-3689	Mice	Decreases VRE numbers in mice feces due to a better recovery of members of the phylum *Bacteroidetes*	[68]
	*L. murinus* Y74 or *L. plantarum* HT121	Mice	Restores gut microbiome diversity and down-regulates host defense-related genes such as defensin α, Apoa1, and RegIII	[12]
	*E. faecalis* X98	Mice	Reduces VRE due to competition	[71]
	*Olsenella* sp.	Mice	Restricts VRE gut colonization	[14]
	A four-strain-consortium named CBBP, consisting of *C. bolteae*, *Blautia producta*, *Bacteroides sartorii*, and *Parabacteroides distasonis*	Mice	Reverses antibiotic-induced susceptibility	[13,72]

* *C.*, *Clostridioides*; *B.*, *Bacteroides*; *E.*, *Enterococcus*; *L.*, different genera of lactobacilli; *S.*, *Salmonella*.

## Data Availability

No new data were created or analyzed in this study. Data sharing is not applicable to this article.

## Abbreviations

The following abbreviations are used in this manuscript:

GI	Gastrointestinal
FMT	Fecal microbiota transplantation
IMT	Intestinal microbiota transplantation
*C. difficile*	*Clostridioides difficile*
*S.* Typhimurium	Serovar Typhimurium
VRE	Vancomycin-resistant *Enterococcus*
CDI	*C. difficile* infection
SCFAs	Short-chain fatty acids
*B. thetaiotaomicron*	*Bacteroides thetaiotaomicron*
ASF	Schaedler flora
SBAs	Secondary bile acids
HDAC3	Histone deacetylase 3
DCA	Deoxycholic acid
LCA	Lithocholic acid
AIs	Autoinducers
T3SS	Type III secretion system
DSFs	Diffusible signal factors
TCA	Taurocholate
MRSA	Methicillin-resistant *Staphylococcus aureus*
ASD	Acute sleep deprivation
ChA	Chlorotonil A

## References

[1] Sender R., Fuchs S., Milo R. (2016). Revised Estimates for the Number of Human and Bacteria Cells in the Body. PLoS Biol..

[2] Ghani R., Chrysostomou D., Roberts L.A., Pandiaraja M., Marchesi J.R., Mullish B.H. (2024). Faecal (or Intestinal) Microbiota Transplant: A Tool for Repairing the Gut Microbiome. Gut Microbes.

[3] Wei Y., Palacios Araya D., Palmer K.L. (2024). Enterococcus Faecium: Evolution, Adaptation, Pathogenesis and Emerging Therapeutics. Nat. Rev. Microbiol..

[4] Millard S.A., Vendrov K.C., Young V.B., Seekatz A.M. (2025). Host Origin of Microbiota Drives Functional Recovery and *Clostridioides difficile* Clearance in Mice. mBio.

[5] Ducarmon Q.R., Grundler F., Le Maho Y., Wilhelmi de Toledo F., Zeller G., Habold C., Mesnage R. (2023). Remodelling of the Intestinal Ecosystem during Caloric Restriction and Fasting. Trends Microbiol..

[6] Liang L., Yang Z., Fu X. (2025). Ecology of the Gut Microbiota and Colonization Resistance: Mechanisms and Therapeutic Implications. FEMS Microbiol. Ecol..

[7] Elzinga J., van der Oost J., de Vos W.M., Smidt H. (2019). The Use of Defined Microbial Communities to Model Host-Microbe Interactions in the Human Gut. Microbiol. Mol. Biol. Rev. MMBR.

[8] Dsouza M., Menon R., Crossette E., Bhattarai S.K., Schneider J., Kim Y.G., Reddy S., Caballero S., Felix C., Cornacchione L. (2022). Colonization of the Live Biotherapeutic Product VE303 and Modulation of the Microbiota and Metabolites in Healthy Volunteers. Cell Host Microbe.

[9] Khanna S., Assi M., Lee C., Yoho D., Louie T., Knapple W., Aguilar H., Garcia-Diaz J., Wang G.P., Berry S.M. (2022). Efficacy and Safety of RBX2660 in PUNCH CD3, a Phase III, Randomized, Double-Blind, Placebo-Controlled Trial with a Bayesian Primary Analysis for the Prevention of Recurrent *Clostridioides difficile* Infection. Drugs.

[10] Liu J., Zhu W., Lessing D.J., Chu W. (2023). Synthetic Microbial Consortia for the Treatment of *Clostridioides difficile* Infection in Mice Model. Microb. Biotechnol..

[11] Muyyarikkandy M.S., Amalaradjou M.A. (2017). *Lactobacillus bulgaricus*, *Lactobacillus rhamnosus* and *Lactobacillus paracasei* Attenuate *Salmonella* Enteritidis, *Salmonella* Heidelberg and *Salmonella* Typhimurium Colonization and Virulence Gene Expression in Vitro. Int. J. Mol. Sci..

[12] Li X., Chu Q., Huang Y., Xiao Y., Song L., Zhu S., Kang Y., Lu S., Xu J., Ren Z. (2019). Consortium of Probiotics Attenuates Colonization of *Clostridioides difficile*. Front. Microbiol..

[13] Kim S.G., Becattini S., Moody T.U., Shliaha P.V., Littmann E.R., Seok R., Gjonbalaj M., Eaton V., Fontana E., Amoretti L. (2019). Microbiota-Derived Lantibiotic Restores Resistance against Vancomycin-Resistant Enterococcus. Nature.

[14] Isaac S., Flor-Duro A., Carruana G., Puchades-Carrasco L., Quirant A., Lopez-Nogueroles M., Pineda-Lucena A., Garcia-Garcera M., Ubeda C. (2022). Microbiome-Mediated Fructose Depletion Restricts Murine Gut Colonization by Vancomycin-Resistant Enterococcus. Nat. Commun..

[15] Cherrak Y., Younes A.A., Perez-Molphe-Montoya E., Maurer L., Yilmaz K., Enz U., Zeder C., Kiefer P., Christen P., Gül E. (2025). Neutrophil Recruitment during Intestinal Inflammation Primes *Salmonella* Elimination by Commensal *E. coli* in a Context-Dependent Manner. Cell Host Microbe.

[16] Jan A., Bayle P., Mohellibi N., Lemoine C., Pepke F., Béguet-Crespel F., Jouanin I., Tremblay-Franco M., Laroche B., Serror P. (2025). A Consortium of Seven Commensal Bacteria Promotes Gut Microbiota Recovery and Strengthens Ecological Barrier against Vancomycin-Resistant Enterococci. Microbiome.

[17] Yoo W., Shealy N.G., Zieba J.K., Torres T.P., Baltagulov M., Thomas J.D., Shelton C.D., McGovern A.G., Foegeding N.J., Olsan E.E. (2024). *Salmonella* Typhimurium Expansion in the Inflamed Murine Gut Is Dependent on Aspartate Derived from ROS-Mediated Microbiota Lysis. Cell Host Microbe.

[18] Gilbert J.A., Azad M.B., Bäckhed F., Blaser M.J., Byndloss M., Chiu C.Y., Chu H., Dugas L.R., Elinav E., Gibbons S.M. (2025). Clinical Translation of Microbiome Research. Nat. Med..

[19] Brown J.M., Hazen S.L. (2017). Targeting of Microbe-Derived Metabolites to Improve Human Health: The next Frontier for Drug Discovery. J. Biol. Chem..

[20] Kim C.H. (2018). Immune Regulation by Microbiome Metabolites. Immunology.

[21] Li Z., Quan G., Jiang X., Yang Y., Ding X., Zhang D., Wang X., Hardwidge P.R., Ren W., Zhu G. (2018). Effects of Metabolites Derived from Gut Microbiota and Hosts on Pathogens. Front. Cell. Infect. Microbiol..

[22] Mehta N., Guzzetta V., Liu K.H., Webster A.S., Adelman M.W., Fitts E.C., Jones D.P., Kraft C.S., Woodworth M.H., Collins J.M. (2026). High-Resolution Metabolomic Analysis of Stool Reveals Expanded Biomarkers of *C. difficile* Colitis and Insights into Pathophysiology. Microbiol. Spectr..

[23] Shi L., Jin L., Huang W. (2023). Bile Acids, Intestinal Barrier Dysfunction, and Related Diseases. Cells.

[24] Gu Q., Yan J., Lou Y., Zhang Z., Li Y., Zhu Z., Liu M., Wu D., Liang Y., Pu J. (2024). Bacteriocins: Curial Guardians of Gastrointestinal Tract. Compr. Rev. Food Sci. Food Saf..

[25] Mukhopadhya I., Louis P. (2025). Gut Microbiota-Derived Short-Chain Fatty Acids and Their Role in Human Health and Disease. Nat. Rev. Microbiol..

[26] Williams L.M., Cao S. (2024). Harnessing and Delivering Microbial Metabolites as Therapeutics via Advanced Pharmaceutical Approaches. Pharmacol. Ther..

[27] Jones K., de Brito C.B., Byndloss M.X. (2025). Metabolic Tug-of-War: Microbial Metabolism Shapes Colonization Resistance against Enteric Pathogens. Cell Chem. Biol..

[28] Fobofou S.A., Savidge T. (2022). Microbial Metabolites: Cause or Consequence in Gastrointestinal Disease?. Am. J. Physiol. Gastrointest. Liver Physiol..

[29] Perl M., Fante M.A., Herfeld K., Scherer J.N., Poeck H., Thiele Orberg E. (2025). Microbiota-Derived Metabolites: Key Modulators of Cancer Immunotherapies. Med.

[30] Jacobson A., Lam L., Rajendram M., Tamburini F., Honeycutt J., Pham T., Van Treuren W., Pruss K., Stabler S.R., Lugo K. (2018). A Gut Commensal-Produced Metabolite Mediates Colonization Resistance to *Salmonella* Infection. Cell Host Microbe.

[31] Shelton C.D., Yoo W., Shealy N.G., Torres T.P., Zieba J.K., Calcutt M.W., Foegeding N.J., Kim D., Kim J., Ryu S. (2022). *Salmonella* Enterica Serovar Typhimurium Uses Anaerobic Respiration to Overcome Propionate-Mediated Colonization Resistance. Cell Rep..

[32] Schofield W.B., Zimmermann-Kogadeeva M., Zimmermann M., Barry N.A., Goodman A.L. (2018). The Stringent Response Determines the Ability of a Commensal Bacterium to Survive Starvation and to Persist in the Gut. Cell Host Microbe.

[33] Nørgaard L.S., Phillips B.L., Hall M.D. (2019). Infection in Patchy Populations: Contrasting Pathogen Invasion Success and Dispersal at Varying Times since Host Colonization. Evol. Lett..

[34] Pamer E.G. (2016). Resurrecting the Intestinal Microbiota to Combat Antibiotic-Resistant Pathogens. Science.

[35] Seekatz A.M., Safdar N., Khanna S. (2022). The Role of the Gut Microbiome in Colonization Resistance and Recurrent *Clostridioides difficile* Infection. Ther. Adv. Gastroenterol..

[36] Lee-Sarwar K.A., Lasky-Su J., Kelly R.S., Litonjua A.A., Weiss S.T. (2020). Metabolome-Microbiome Crosstalk and Human Disease. Metabolites.

[37] Olsan E.E., Byndloss M.X., Faber F., Rivera-Chávez F., Tsolis R.M., Bäumler A.J. (2017). Colonization Resistance: The Deconvolution of a Complex Trait. J. Biol. Chem..

[38] Li N., Guo X. (2026). The Gut Microbiota and Host Immunity Synergistically Orchestrate Colonization Resistance. Gut Microbes.

[39] Khan I., Bai Y., Zha L., Ullah N., Ullah H., Shah S.R.H., Sun H., Zhang C. (2021). Mechanism of the Gut Microbiota Colonization Resistance and Enteric Pathogen Infection. Front. Cell. Infect. Microbiol..

[40] Buffie C.G., Pamer E.G. (2013). Microbiota-Mediated Colonization Resistance against Intestinal Pathogens. Nat. Rev. Immunol..

[41] Sandhu B.K., McBride S.M. (2018). *Clostridioides* *difficile*. Trends Microbiol..

[42] Khanna S., Voth E. (2023). Therapeutics for *Clostridioides difficile* Infection: Molecules and Microbes. Expert Rev. Gastroenterol. Hepatol..

[43] Tejada J.N., Walters W.A., Wang Y., Kordahi M., Chassaing B., Pickard J., Nunez G., Ley R., Gewirtz A.T. (2024). Prevention and Cure of Murine *C. difficile* Infection by a Lachnospiraceae Strain. Gut Microbes.

[44] Spinler J.K., Auchtung J., Brown A., Boonma P., Oezguen N., Ross C.L., Luna R.A., Runge J., Versalovic J., Peniche A. (2017). Next-Generation Probiotics Targeting *Clostridium difficile* through Precursor-Directed Antimicrobial Biosynthesis. Infect. Immun..

[45] Li X., Kang Y., Huang Y., Xiao Y., Song L., Lu S., Ren Z. (2021). A Strain of *Bacteroides thetaiotaomicron* Attenuates Colonization of *Clostridioides difficile* and Affects Intestinal Microbiota and Bile Acids Profile in a Mouse Model. Biomed. Pharmacother..

[46] Elahi M., Nakayama-Imaohji H., Hashimoto M., Tada A., Yamasaki H., Nagao T., Kuwahara T. (2021). The Human Gut Microbe *Bacteroides thetaiotaomicron* Suppresses Toxin Release from *Clostridium difficile* by Inhibiting Autolysis. Antibiotics.

[47] Mills J.P., Rao K., Young V.B. (2018). Probiotics for Prevention of *Clostridium difficile* Infection. Curr. Opin. Gastroenterol..

[48] Hagihara M., Ariyoshi T., Kuroki Y., Eguchi S., Higashi S., Mori T., Nonogaki T., Iwasaki K., Yamashita M., Asai N. (2021). *Clostridium butyricum* Enhances Colonization Resistance against *Clostridioides difficile* by Metabolic and Immune Modulation. Sci. Rep..

[49] Saenz C., Fang Q., Gnanasekaran T., Trammell S.A.J., Buijink J.D.A., Pisano P., Wierer M., Moens F., Lengger B., Brejnrod A. (2023). *Clostridium scindens* Secretome Suppresses Virulence Gene Expression of *Clostridioides difficile* in a Bile Acid-Independent Manner. Microbiol. Spectr..

[50] Seekatz A.M., Theriot C.M., Rao K., Chang Y.M., Freeman A.E., Kao J.Y., Young V.B. (2018). Restoration of Short Chain Fatty Acid and Bile Acid Metabolism Following Fecal Microbiota Transplantation in Patients with Recurrent *Clostridium difficile* Infection. Anaerobe.

[51] Tian H., Cui J., Ye C., Zhao J., Yang B., Xu Y., Ji S., Wang L., Lv X., Ma C. (2023). Depletion of Butyrate-Producing Microbes of the Firmicutes Predicts Nonresponse to FMT Therapy in Patients with Recurrent *Clostridium difficile* Infection. Gut Microbes.

[52] Cassir N., Couturier J., Delannoy J., Wolfromm A., Kassis Chikhani N., Barbut F. (2024). Gut Microbiota Composition and *Clostridioides difficile* Infection: The Potential Protective Role of *Faecalibacterium prausnitzii*. Gut Microbes Rep..

[53] Markey L., Shaban L., Green E.R., Lemon K.P., Mecsas J., Kumamoto C.A. (2018). Pre-Colonization with the Commensal Fungus Candida Albicans Reduces Murine Susceptibility to *Clostridium difficile* Infection. Gut Microbes.

[54] Lacotte P.A., Simons A., Bouttier S., Malet-Villemagne J., Nicolas V., Janoir C. (2022). Inhibition of in Vitro *Clostridioides difficile* Biofilm Formation by the Probiotic Yeast *Saccharomyces boulardii* CNCM I-745 through Modification of the Extracellular Matrix Composition. Microorganisms.

[55] Wombwell E. (2023). *Saccharomyces boulardii* Prophylaxis for Targeted Antibiotics and Infectious Indications to Reduce Healthcare Facility-Onset *Clostridioides difficile* Infection. Microbes Infect..

[56] Feuerstadt P., Louie T.J., Lashner B., Wang E.E.L., Diao L., Bryant J.A., Sims M., Kraft C.S., Cohen S.H., Berenson C.S. (2022). SER-109, an Oral Microbiome Therapy for Recurrent *Clostridioides difficile* Infection. N. Engl. J. Med..

[57] Rangan K.J., Pedicord V.A., Wang Y.C., Kim B., Lu Y., Shaham S., Mucida D., Hang H.C. (2016). A Secreted Bacterial Peptidoglycan Hydrolase Enhances Tolerance to Enteric Pathogens. Science.

[58] Pedicord V.A., Lockhart A.A.K., Rangan K.J., Craig J.W., Loschko J., Rogoz A., Hang H.C., Mucida D. (2016). Exploiting a Host-Commensal Interaction to Promote Intestinal Barrier Function and Enteric Pathogen Tolerance. Sci. Immunol..

[59] Sassone-Corsi M., Nuccio S.P., Liu H., Hernandez D., Vu C.T., Takahashi A.A., Edwards R.A., Raffatellu M. (2016). Microcins Mediate Competition among Enterobacteriaceae in the Inflamed Gut. Nature.

[60] Sassone-Corsi M., Chairatana P., Zheng T., Perez-Lopez A., Edwards R.A., George M.D., Nolan E.M., Raffatellu M. (2016). Siderophore-Based Immunization Strategy to Inhibit Growth of Enteric Pathogens. Proc. Natl. Acad. Sci. USA.

[61] Eberl C., Weiss A.S., Jochum L.M., Durai Raj A.C., Ring D., Hussain S., Herp S., Meng C., Kleigrewe K., Gigl M. (2021). *E. coli* Enhance Colonization Resistance against *Salmonella* Typhimurium by Competing for Galactitol, a Context-Dependent Limiting Carbon Source. Cell Host Microbe.

[62] Wang B., Peng X., Zhou X., Jin X., Siddique A., Yao J., Zhang H., Li W., Li Y., Yue M. (2025). *Lacticaseibacillus rhamnosus* P118 Enhances Host Tolerance to *Salmonella* Infection by Promoting Microbe-Derived Indole Metabolites. eLife.

[63] Gao X., Zhou J., Yan K., Guan Y., Xiang J., Liu Y., Yu H., Wang J., Li Y., Xu Y. (2026). *Lactobacillus rhamnosus* Confers Protection against Enteropathogenic Bacteria by Enhancing Mucosal Immunity and Epithelial Barrier Function. Front. Cell. Infect. Microbiol..

[64] Herp S., Brugiroux S., Garzetti D., Ring D., Jochum L.M., Beutler M., Eberl C., Hussain S., Walter S., Gerlach R.G. (2019). *Mucispirillum schaedleri* Antagonizes *Salmonella* Virulence to Protect Mice against Colitis. Cell Host Microbe.

[65] Brugiroux S., Beutler M., Pfann C., Garzetti D., Ruscheweyh H.J., Ring D., Diehl M., Herp S., Lötscher Y., Hussain S. (2016). Genome-Guided Design of a Defined Mouse Microbiota That Confers Colonization Resistance against *Salmonella* Enterica Serovar Typhimurium. Nat. Microbiol..

[66] Thiemann S., Smit N., Roy U., Lesker T.R., Gálvez E.J.C., Helmecke J., Basic M., Bleich A., Goodman A.L., Kalinke U. (2017). Enhancement of IFNγ Production by Distinct Commensals Ameliorates *Salmonella*-Induced Disease. Cell Host Microbe.

[67] Meinen-Jochum J., Ott L.C., Mellata M. (2023). Segmented Filamentous Bacteria-Based Treatment to Elicit Protection against Enterobacteriaceae in Layer Chickens. Front. Microbiol..

[68] Crouzet L., Derrien M., Cherbuy C., Plancade S., Foulon M., Chalin B., van Hylckama Vlieg J.E.T., Grompone G., Rigottier-Gois L., Serror P. (2018). *Lactobacillus paracasei* CNCM I-3689 Reduces Vancomycin-Resistant Enterococcus Persistence and Promotes Bacteroidetes Resilience in the Gut Following Antibiotic Challenge. Sci. Rep..

[69] Li X., Song L., Zhu S., Xiao Y., Huang Y., Hua Y., Chu Q., Ren Z. (2019). Two Strains of Lactobacilli Effectively Decrease the Colonization of VRE in a Mouse Model. Front. Cell. Infect. Microbiol..

[70] Fan F.H., Lin Y.J., Chen J.H., Fang S.B. (2026). Efficacy of Probiotics in Vancomycin-Resistant Enterococci Decolonization: A Meta-Analysis of Randomized Clinical Trials. Pediatr. Neonatol..

[71] Ben-Assa N., Naddaf R., Carasso S., Dagan O., Sason A., Gefen T., Geva-Zatorsky N. (2026). Intra-Species Competition Combats Vancomycin-Resistant Enterococci. Gut Microbes.

[72] Caballero S., Kim S., Carter R.A., Leiner I.M., Sušac B., Miller L., Kim G.J., Ling L., Pamer E.G. (2017). Cooperating Commensals Restore Colonization Resistance to Vancomycin-Resistant Enterococcus Faecium. Cell Host Microbe.

[73] Kellogg T.D., Ceglia S., Mortzfeld B.M., Tanna T.M., Zeamer A.L., Mancini M.R., Foley S.E., Ward D.V., Bhattarai S.K., McCormick B.A. (2025). Succinate-Producing Microbiota Drives Tuft Cell Hyperplasia to Protect Against *Clostridioides difficile*. J. Exp. Med..

[74] Louis P., Flint H.J. (2017). Formation of Propionate and Butyrate by the Human Colonic Microbiota. Environ. Microbiol..

[75] Tsugawa H., Kabe Y., Kanai A., Sugiura Y., Hida S., Taniguchi S., Takahashi T., Matsui H., Yasukawa Z., Itou H. (2020). Short-Chain Fatty Acids Bind to Apoptosis-Associated Speck-like Protein to Activate Inflammasome Complex to Prevent *Salmonella* Infection. PLoS Biol..

[76] Gregory A.L., Pensinger D.A., Hryckowian A.J. (2021). A Short Chain Fatty Acid-Centric View of *Clostridioides difficile* Pathogenesis. PLoS Pathog..

[77] Beier R.C., Harvey R.B., Poole T.L., Hume M.E., Crippen T.L., Highfield L.D., Alali W.Q., Andrews K., Anderson R.C., Nisbet D.J. (2019). Interactions of Organic Acids with Vancomycin-Resistant Enterococcus Faecium Isolated from Community Wastewater in Texas. J. Appl. Microbiol..

[78] Shi Y., Xu M., Pan S., Gao S., Ren J., Bai R., Li H., He C., Zhao S., Shi Z. (2021). Induction of the Apoptosis, Degranulation and IL-13 Production of Human Basophils by Butyrate and Propionate via Suppression of Histone Deacetylation. Immunology.

[79] Kang T., Yim D., Kim S.S., Baek K.H., Kim H.J., Jo C. (2022). Effect of Plasma-Activated Acetic Acid on Inactivation of *Salmonella* Typhimurium and Quality Traits on Chicken Meats. Poult. Sci..

[80] Lu L.F., Kim D.H., Lee I.H., Hong J., Zhang P., Yoon I.N., Hwang J.S., Kim H. (2016). Potassium Acetate Blocks *Clostridium difficile* Toxin A-Induced Microtubule Disassembly by Directly Inhibiting Histone Deacetylase 6, Thereby Ameliorating Inflammatory Responses in the Gut. J. Microbiol. Biotechnol..

[81] Fachi J.L., Sécca C., Rodrigues P.B., Mato F.C.P., Di Luccia B., Felipe J.S., Pral L.P., Rungue M., Rocha V.M., Sato F.T. (2020). Acetate Coordinates Neutrophil and ILC3 Responses against *C. difficile* through FFAR2. J. Exp. Med..

[82] Meng F., Zhao H., Nie T., Lu F., Zhang C., Lu Y., Lu Z. (2021). Acetate Activates Lactobacillus Bacteriocin Synthesis by Controlling Quorum Sensing. Appl. Environ. Microbiol..

[83] Langfeld L.Q., Du K., Bereswill S., Heimesaat M.M. (2021). A Review of the Antimicrobial and Immune-Modulatory Properties of the Gut Microbiota-Derived Short Chain Fatty Acid Propionate—What Is New?. Eur. J. Microbiol. Immunol..

[84] Bajic D., Niemann A., Hillmer A.K., Mejias-Luque R., Bluemel S., Docampo M., Funk M.C., Tonin E., Boutros M., Schnabl B. (2020). Gut Microbiota-Derived Propionate Regulates the Expression of Reg3 Mucosal Lectins and Ameliorates Experimental Colitis in Mice. J. Crohn’s Colitis.

[85] Adesina P.A., Saeki I., Yamamoto Y., Suzuki T. (2023). N-Butyrate Increases Heat Shock Protein 70 through Heat Shock Factor 1 and AMP-Activated Protein Kinase Pathways in Human Intestinal Caco-2 Cells. Arch. Biochem. Biophys..

[86] Tang H., Zhan Z., Liu X., Zhang Y., Huang X., Xu M. (2023). Propionate Reduces the Viability of *Salmonella enterica Serovar* Typhi in Macrophages by Propionylation of PhoP K102. Microb. Pathog..

[87] Jeong S., Lee Y., Yun C.H., Park O.J., Han S.H. (2019). Propionate, Together with Triple Antibiotics, Inhibits the Growth of Enterococci. J. Microbiol..

[88] Kalkan A.E., BinMowyna M.N., Raposo A., Ahmad M.F., Ahmed F., Otayf A.Y., Carrascosa C., Saraiva A., Karav S. (2025). Beyond the Gut: Unveiling Butyrate’s Global Health Impact through Gut Health and Dysbiosis-Related Conditions: A Narrative Review. Nutrients.

[89] Schulthess J., Pandey S., Capitani M., Rue-Albrecht K.C., Arnold I., Franchini F., Chomka A., Ilott N.E., Johnston D.G.W., Pires E. (2019). The Short Chain Fatty Acid Butyrate Imprints an Antimicrobial Program in Macrophages. Immunity.

[90] Wang S., Xiang L., Li F., Deng W., Lv P., Chen Y. (2023). Butyrate Protects against *Clostridium difficile* Infection by Regulating Bile Acid Metabolism. Microbiol. Spectr..

[91] Pensinger D.A., Fisher A.T., Dobrila H.A., Van Treuren W., Gardner J.O., Higginbottom S.K., Carter M.M., Schumann B., Bertozzi C.R., Anikst V. (2023). Butyrate Differentiates Permissiveness to *Clostridioides difficile* Infection and Influences Growth of Diverse *C. difficile* Isolates. Infect. Immun..

[92] Baldassare M.A., Bhattacharjee D., Coles J.D., Nelson S., McCollum C.A., Seekatz A.M. (2023). Butyrate Enhances *Clostridioides difficile* Sporulation in Vitro. J. Bacteriol..

[93] Liu J., Zhu W., Qin N., Ren X., Xia X. (2022). Propionate and Butyrate Inhibit Biofilm Formation of *Salmonella* Typhimurium Grown in Laboratory Media and Food Models. Foods.

[94] Park O.J., Ha Y.E., Sim J.R., Lee D., Lee E.H., Kim S.Y., Yun C.H., Han S.H. (2023). Butyrate Potentiates Enterococcus Faecalis Lipoteichoic Acid-Induced Inflammasome Activation via Histone Deacetylase Inhibition. Cell Death Discov..

[95] Winston J.A., Theriot C.M. (2020). Diversification of Host Bile Acids by Members of the Gut Microbiota. Gut Microbes.

[96] Guzior D.V., Quinn R.A. (2021). Review: Microbial Transformations of Human Bile Acids. Microbiome.

[97] Aguirre A.M., Sorg J.A. (2022). Gut Associated Metabolites and Their Roles in *Clostridioides difficile* Pathogenesis. Gut Microbes.

[98] Thanissery R., Winston J.A., Theriot C.M. (2017). Inhibition of Spore Germination, Growth, and Toxin Activity of Clinically Relevant *C. difficile* Strains by Gut Microbiota Derived Secondary Bile Acids. Anaerobe.

[99] Kisthardt S.C., Thanissery R., Pike C.M., Foley M.H., Theriot C.M. (2023). The Microbial-Derived Bile Acid Lithocholate and Its Epimers Inhibit *Clostridioides difficile* Growth and Pathogenicity While Sparing Members of the Gut Microbiota. J. Bacteriol..

[100] Studer N., Desharnais L., Beutler M., Brugiroux S., Terrazos M.A., Menin L., Schürch C.M., McCoy K.D., Kuehne S.A., Minton N.P. (2016). Functional Intestinal Bile Acid 7α-Dehydroxylation by *Clostridium scindens* Associated with Protection from *Clostridium difficile* Infection in a Gnotobiotic Mouse Model. Front. Cell. Infect. Microbiol..

[101] Eade C.R., Hung C.C., Bullard B., Gonzalez-Escobedo G., Gunn J.S., Altier C. (2016). Bile Acids Function Synergistically to Repress Invasion Gene Expression in *Salmonella* by Destabilizing the Invasion Regulator HilD. Infect. Immun..

[102] Yang X., Stein K.R., Hang H.C. (2023). Anti-Infective Bile Acids Bind and Inactivate a *Salmonella* Virulence Regulator. Nat. Chem. Biol..

[103] Tremblay S., Romain G., Roux M., Chen X.L., Brown K., Gibson D.L., Ramanathan S., Menendez A. (2017). Bile Acid Administration Elicits an Intestinal Antimicrobial Program and Reduces the Bacterial Burden in Two Mouse Models of Enteric Infection. Infect. Immun..

[104] McKenney P.T., Yan J., Vaubourgeix J., Becattini S., Lampen N., Motzer A., Larson P.J., Dannaoui D., Fujisawa S., Xavier J.B. (2019). Intestinal Bile Acids Induce a Morphotype Switch in Vancomycin-Resistant Enterococcus That Facilitates Intestinal Colonization. Cell Host Microbe.

[105] Chassaing B., Cascales E. (2018). Antibacterial Weapons: Targeted Destruction in the Microbiota. Trends Microbiol..

[106] Schwemmlein N., Pippel J., Gazdag E.M., Blankenfeldt W. (2018). Crystal Structures of R-Type Bacteriocin Sheath and Tube Proteins CD1363 and CD1364 from *Clostridium difficile* in the Pre-Assembled State. Front. Microbiol..

[107] Son Y.J., Kim Y.R., Oh S.H., Jung S., Ciufolini M.A., Hwang H.J., Kwak J.H., Pai H. (2022). Micrococcin P2 Targets *Clostridioides difficile*. J. Nat. Prod..

[108] O’Reilly C., O’Connor P.M., O’Sullivan Ó., Rea M.C., Hill C., Ross R.P. (2022). Impact of Nisin on *Clostridioides difficile* and Microbiota Composition in a Faecal Fermentation Model of the Human Colon. J. Appl. Microbiol..

[109] Sandiford S.K. (2019). Current Developments in Lantibiotic Discovery for Treating *Clostridium difficile* Infection. Expert Opin. Drug Discov..

[110] Reinseth I.S., Ovchinnikov K.V., Tønnesen H.H., Carlsen H., Diep D.B. (2020). The Increasing Issue of Vancomycin-Resistant Enterococci and the Bacteriocin Solution. Probiotics Antimicrob. Proteins.

[111] Yüksel F.N., Buzrul S., Akçelik M., Akçelik N. (2018). Inhibition and Eradication of *Salmonella* Typhimurium Biofilm Using P22 Bacteriophage, EDTA and Nisin. Biofouling.

[112] Sengkhui S., Klubthawee N., Aunpad R. (2023). A Novel Designed Membrane-Active Peptide for the Control of Foodborne *Salmonella* Enterica Serovar Typhimurium. Sci. Rep..

[113] Cleary J.L., Condren A.R., Zink K.E., Sanchez L.M. (2017). Calling All Hosts: Bacterial Communication in Situ. Chem.

[114] Kohli N., Crisp Z., Riordan R., Li M., Alaniz R.C., Jayaraman A. (2018). The Microbiota Metabolite Indole Inhibits *Salmonella* Virulence: Involvement of the PhoPQ Two-Component System. PLoS ONE.

[115] Nieves K.M., Flannigan K.L., Hughes E., Stephens M., Thorne A.J., Delanne-Cuménal A., Strayer K., Kola-Ilesanmi D., Wickramasinghe S., Mirzadzar N. (2025). Indole-3-Propionic Acid Protects Medium-Diversity Colitic Mice via Barrier Enhancement Preferentially over Anti-Inflammatory Effects. Am. J. Physiol. Gastrointest. Liver Physiol..

[116] Wlodarska M., Luo C., Kolde R., d’Hennezel E., Annand J.W., Heim C.E., Krastel P., Schmitt E.K., Omar A.S., Creasey E.A. (2017). Indoleacrylic Acid Produced by Commensal *Peptostreptococcus* Species Suppresses Inflammation. Cell Host Microbe.

[117] Yuan W., Yu Z., Song W., Li Y., Fang Z., Zhu B., Li X., Wang H., Hong W., Sun N. (2019). Indole-Core-Based Novel Antibacterial Agent Targeting FtsZ. Infect. Drug Resist..

[118] Darkoh C., Plants-Paris K., Bishoff D., DuPont H.L. (2019). *Clostridium difficile* Modulates the Gut Microbiota by Inducing the Production of Indole, an Interkingdom Signaling and Antimicrobial Molecule. mSystems.

[119] Li S., Sun H., Li J., Zhao Y., Wang R., Xu L., Duan C., Li J., Wang Z., Liu Q. (2022). Autoinducer-2 and Bile Salts Induce c-Di-GMP Synthesis to Repress the T3SS via a T3SS Chaperone. Nat. Commun..

[120] Lallement C., Goldring W.P.D., Jelsbak L. (2023). Global Transcriptomic Response of the AI-3 Isomers 3,5-DPO and 3,6-DPO in *Salmonella* Typhimurium. Arch. Microbiol..

[121] Piewngam P., Zheng Y., Nguyen T.H., Dickey S.W., Joo H.S., Villaruz A.E., Glose K.A., Fisher E.L., Hunt R.L., Li B. (2018). Pathogen Elimination by Probiotic *Bacillus* via Signalling Interference. Nature.

[122] Chowdhury R., Pavinski Bitar P.D., Chapman H.M., Altier C. (2023). *Salmonella* Invasion Is Controlled by Competition among Intestinal Chemical Signals. mBio.

[123] Bosire E.M., Eade C.R., Schiltz C.J., Reid A.J., Troutman J., Chappie J.S., Altier C. (2020). Diffusible Signal Factors Act through AraC-Type Transcriptional Regulators as Chemical Cues to Repress Virulence of Enteric Pathogens. Infect. Immun..

[124] Miller A.L., Nicastro L.K., Bessho S., Grando K., White A.P., Zhang Y., Queisser G., Buttaro B.A., Tükel Ç. (2021). Nitrate Is an Environmental Cue in the Gut for *Salmonella* Enterica Serovar Typhimurium Biofilm Dispersal through Curli Repression and Flagellum Activation via Cyclic-Di-GMP Signaling. mBio.

[125] Caballero-Flores G., Pickard J.M., Núñez G. (2023). Microbiota-Mediated Colonization Resistance: Mechanisms and Regulation. Nat. Rev. Microbiol..

[126] Metzendorf N.G., Lange L.M., Lainer N., Schlüter R., Dittmann S., Paul L.S., Troitzsch D., Sievers S. (2022). Destination and Specific Impact of Different Bile Acids in the Intestinal Pathogen *Clostridioides difficile*. Front. Microbiol..

[127] Repoila F., Le Bohec F., Guérin C., Lacoux C., Tiwari S., Jaiswal A.K., Santana M.P., Kennedy S.P., Quinquis B., Rainteau D. (2022). Adaptation of the Gut Pathobiont Enterococcus Faecalis to Deoxycholate and Taurocholate Bile Acids. Sci. Rep..

[128] McMillan A.S., Theriot C.M. (2024). Bile Acids Impact the Microbiota, Host, and *C. difficile* Dynamics Providing Insight into Mechanisms of Efficacy of FMTs and Microbiota-Focused Therapeutics. Gut Microbes.

[129] Harnvoravongchai P., Mattiello S.P., Amabat A., C. P. J., Faisal S.M., Kaushik R.S., Scaria J. (2026). Gut Commensal Bifidobacterium Longum Confers Resistance to *Salmonella* Typhimurium and Shigella Flexneri in a Caenorhabditis Elegans Model. Microbiol. Spectr..

[130] Peng M., Tabashsum Z., Patel P., Bernhardt C., Biswas C., Meng J., Biswas D. (2020). Prevention of Enteric Bacterial Infections and Modulation of Gut Microbiota with Conjugated Linoleic Acids Producing Lactobacillus in Mice. Gut Microbes.

[131] Carolak E., Czajkowska J., Waszczuk W., Dutkiewicz A., Kedzierska A.E., Grzymajlo K. (2026). Genetically Engineered Probiotic *E. coli* Nissle 1917 Enhances Protection against *Salmonella* via Increased Adhesion and Systemic T-Cell Responses. npj Biofilms Microbiomes.

[132] Deehan E.C., Mocanu V., Madsen K.L. (2024). Effects of Dietary Fibre on Metabolic Health and Obesity. Nat. Rev. Gastroenterol. Hepatol..

[133] Dong Y., Han M., Fei T., Liu H., Gai Z. (2024). Utilization of Diverse Oligosaccharides for Growth by Bifidobacterium and Lactobacillus Species and Their in Vitro Co-Cultivation Characteristics. Int. Microbiol. Off. J. Span. Soc. Microbiol..

[134] Teichmann J., Cockburn D.W. (2021). In Vitro Fermentation Reveals Changes in Butyrate Production Dependent on Resistant Starch Source and Microbiome Composition. Front. Microbiol..

[135] Ahmed S., Roberts K.D., McCormick T.S., Ghannoum M.A. (2026). Improving Growth Dynamics of *Faecalibacterium prausnitzii* by Exposure to Prebiotics. Int. J. Mol. Sci..

[136] Jung S., Bae H., Song W.S., Jang C. (2022). Dietary Fructose and Fructose-Induced Pathologies. Annu. Rev. Nutr..

[137] Holeček M. (2026). Serotonin, Kynurenine, and Indole Pathways of Tryptophan Metabolism in Humans in Health and Disease. Nutrients.

[138] Van Buiten C.B., Seitz V.A., Metcalf J.L., Raskin I. (2024). Dietary Polyphenols Support *Akkermansia muciniphila* Growth via Mediation of the Gastrointestinal Redox Environment. Antioxidants.

[139] Rodríguez-Daza M.C., Boeren S., Tytgat H.L.P., Desjardins Y., de Vos W.M. (2025). *Akkermansia muciniphila* MucT Harnesses Dietary Polyphenols as Xenosiderophores for Enhanced Iron Uptake. Nat. Commun..

[140] Yao Q., Zhang W., Wang Y., Shi L., Zhao Y., Liang J., Zhao Y., Kang J., Zheng X., Guo R. (2025). *Lactobacillus plantarum* and Galacto-Oligosaccharides Synbiotic Relieve Irritable Bowel Syndrome by Reshaping Gut Microbiota and Attenuating Mast Cell Hyperactivation. Nutrients.

[141] Zhang G., Zhang Y., Zheng B., Zeng H., Zheng Y. (2026). Resistant Starch-Driven Remodeling of Gut Microbiota and Metabolite Profiles Enhanced by Sodium Butyrate Supplementation. Food Biosci..

[142] Zou B., Zhao D., Zhou S., Kang J.X., Wang B. (2025). Insight into the Effects of Omega-3 Fatty Acids on Gut Microbiota: Impact of a Balanced Tissue Omega-6/Omega-3 Ratio. Front. Nutr..

[143] Ma B., Wang D., Chen X., Wang Q., Zhang T., Wen R., Yang M., Li C., Lei C., Wang H. (2024). Dietary α-Linolenic Acid Supplementation Enhances Resistance to *Salmonella* Typhimurium Challenge in Chickens by Altering the Intestinal Mucosal Barrier Integrity and Cecal Microbes. Microbiol. Res..

[144] Kim D., Joe H.I., Bae J.W., Wu G.D., Compher C.W., Koo H. (2026). Fermented Food Microbiome: Influence on Oral and Gut Microbiota, and Human Health. Nat. Rev. Microbiol..

[145] Özkul C., Yalınay M., Karakan T. (2019). Islamic Fasting Leads to an Increased Abundance of *Akkermansia muciniphila* and Bacteroides Fragilis Group: A Preliminary Study on Intermittent Fasting. Turk. J. Gastroenterol. Off. J. Turk. Soc. Gastroenterol..

[146] Ashique S., Debnath B., Mojgani N., Tariq M., Haider T., Shorog E., Yasmin S., Islam A., Sharma H., Hussain M.S. (2025). Gut Microbiota Modulation and Health Benefits of Various Fasting Regimens. Curr. Res. Biotechnol..

[147] von Schwartzenberg R.J., Bisanz J.E., Lyalina S., Spanogiannopoulos P., Ang Q.Y., Cai J., Dickmann S., Friedrich M., Liu S.Y., Collins S.L. (2021). Caloric Restriction Disrupts the Microbiota and Colonization Resistance. Nature.

[148] Pickard J.M., Núñez G. (2019). Pathogen Colonization Resistance in the Gut and Its Manipulation for Improved Health. Am. J. Pathol..

[149] Ross F.C., Patangia D., Grimaud G., Lavelle A., Dempsey E.M., Ross R.P., Stanton C. (2024). The Interplay between Diet and the Gut Microbiome: Implications for Health and Disease. Nat. Rev. Microbiol..

[150] Garcia-Mantrana I., Selma-Royo M., Alcantara C., Collado M.C. (2018). Shifts on Gut Microbiota Associated to Mediterranean Diet Adherence and Specific Dietary Intakes on General Adult Population. Front. Microbiol..

[151] Pickard J.M., Porwollik S., Caballero-Flores G., Caruso R., Fukuda S., Soga T., Inohara N., McClelland M., Núñez G. (2025). Dietary Amino Acids Regulate *Salmonella* Colonization via Microbiota-Dependent Mechanisms in the Mouse Gut. Nat. Commun..

[152] Erickson D., Chua M., Sheneman K.R., Hernandez L., Collins J. (2025). A Sucrose-Rich Diet Promotes *Clostridioides difficile* Carriage without Prior Antibiotics and Significantly Exacerbates Both Acute Disease and Long-Term Colonization. Gut Microbes.

[153] Eraghieh Farahani H., Pourhajibagher M., Asgharzadeh S., Bahador A. (2026). Postbiotics: Novel Modulators of Gut Health, Metabolism, and Their Mechanisms of Action. Probiotics Antimicrob. Proteins.

[154] Liang S., Zhao D., Liu X., Liu B., Li Y. (2025). The Stomach, Small Intestine, and Colon-Specific Gastrointestinal Tract Delivery Systems for Bioactive Nutrients. Adv. Colloid Interface Sci..

[155] Chen Y., Pan R., Mei L., Tian P., Wang L., Zhao J., Chen W., Wang G. (2023). Colon-Targeted Delivery of Indole Acetic Acid Helps Regulate Gut Motility by Activating the AHR Signaling Pathway. Nutrients.

[156] Pattapulavar V., Ramanujam S., Kini B., Christopher J.G. (2025). Probiotic-Derived Postbiotics: A Perspective on next-Generation Therapeutics. Front. Nutr..

[157] Liu S., Loo Y.T., Li Z., Ng K. (2023). Alginate-Inulin-Chitosan Based Microspheres Alter Metabolic Fate of Encapsulated Quercetin, Promote Short Chain Fatty Acid Production, and Modulate Pig Gut Microbiota. Food Chem..

[158] Li N., Niu L., Liu Y., Wang Y., Su X., Xu C., Sun Z., Guo H., Gong J., Shen S. (2024). Taking SCFAs Produced by Lactobacillus Reuteri Orally Reshapes Gut Microbiota and Elicits Antitumor Responses. J. Nanobiotechnol..

[159] Wang J.L., Yeh C.H., Huang S.H., Wu L.S., Chen M.C. (2024). Effects of Resistant-Starch-Encapsulated Probiotic Cocktail on Intestines Damaged by 5-Fluorouracil. Biomedicines.

[160] Ji C., Li D., Liang Y., Luo Y. (2025). Co-Encapsulation of Probiotics with Functional Components: Design Strategies, Synergistic Mechanisms, Biomedical Applications, and Challenges for Industrialization. J. Mater. Chem. B.

[161] Hijová E. (2024). Postbiotics as Metabolites and Their Biotherapeutic Potential. Int. J. Mol. Sci..

[162] Geldart K.G., Kommineni S., Forbes M. (2018). Engineered *E. coli* Nissle 1917 for the Reduction of Vancomycin-Resistant Enterococcus in the Intestinal Tract. Bioeng. Transl. Med..

[163] Xiang Y., Peng H., Li Y., Wen H., Li N., Liu X., Yuan G., Shi Y., Huang S. (2026). Cellular Membrane Protein MipA from *E. coli* Nissle 1917 Protects against *Salmonella* Infection. ISME J..

[164] Pereira S.M.S., José V.P.B.S., da Silva A., Martins K.V.C., Leite L.B., Forte P., Natali A.J., Martino H.S.D., Lucia C.M.D., Bressan J. (2025). Can Physical Exercise Modify Intestinal Integrity and Gut Microbiota Composition? A Systematic Review of in Vivo Studies. Biol. Sport.

[165] Hoseini R., Rahim H.A., Saifalddin D.L., Kareem D.A., Fatah A.M. (2025). Exercise Intensity-Mediated Regulation of Gut Epithelial Cells and Immune Function in Gut Microbiota Dysbiosis. J. Transl. Med..

[166] Xu T., Fang D., Xu T., Tao X., Wang Z., Liu Y. (2025). Exercise-Driven Gut Microbiota Alterations Enhance Colonization Resistance against Methicillin-Resistant Staphylococcus Aureus. Cell Rep..

[167] Fang D., Xu T., Sun J., Shi J., Li F., Yin Y., Wang Z., Liu Y. (2023). Nicotinamide Mononucleotide Ameliorates Sleep Deprivation-Induced Gut Microbiota Dysbiosis and Restores Colonization Resistance against Intestinal Infections. Adv. Sci..

[168] Yang D.F., Huang W.C., Wu C.W., Huang C.Y., Yang Y.S.H., Tung Y.T. (2023). Acute Sleep Deprivation Exacerbates Systemic Inflammation and Psychiatry Disorders through Gut Microbiota Dysbiosis and Disruption of Circadian Rhythms. Microbiol. Res..

[169] Supasitdikul T., Mazariegos J.R.R., Nhat N.N., Tung Y.T., Yang D.F., Lee L.J., Gunawan S.P., Chen Y.C. (2026). Sleep Deprivation Alters Gut Microbiome Diversity and Taxonomy: A Systematic Review and Meta-Analysis of Human and Rodent Studies. J. Sleep Res..

[170] Pohl K., Moodley P., Dhanda A.D. (2021). Alcohol’s Impact on the Gut and Liver. Nutrients.

[171] Baek S.M., Kim T.U., Lee Y.J., Lee S.W., Yim J.H., Kim W.J., Kim H.Y., Kang K.K., Kim S.D., Park S.J. (2023). Disrupted Intestinal Mucosal Barrier Mediated by Alcohol Consumption Aggravates Systemic Microplastic Accumulation. Ecotoxicol. Environ. Saf..

[172] Shen M., Zhao H., Han M., Su L., Cui X., Li D., Liu L., Wang C., Yang F. (2024). Alcohol-Induced Gut Microbiome Dysbiosis Enhances the Colonization of Klebsiella Pneumoniae on the Mouse Intestinal Tract. mSystems.

[173] Kuo C.H., Wu L.L., Chen H.P., Yu J., Wu C.Y. (2024). Direct Effects of Alcohol on Gut-Epithelial Barrier: Unraveling the Disruption of Physical and Chemical Barrier of the Gut-Epithelial Barrier That Compromises the Host-Microbiota Interface upon Alcohol Exposure. J. Gastroenterol. Hepatol..

[174] Sosnowski K., Przybyłkowski A. (2024). Ethanol-Induced Changes to the Gut Microbiome Compromise the Intestinal Homeostasis: A Review. Gut Microbes.

[175] Yip A.Y.G., King O.G., Omelchenko O., Kurkimat S., Horrocks V., Mostyn P., Danckert N., Ghani R., Satta G., Jauneikaite E. (2023). Antibiotics Promote Intestinal Growth of Carbapenem-Resistant Enterobacteriaceae by Enriching Nutrients and Depleting Microbial Metabolites. Nat. Commun..

[176] King O.G., Yip A.Y.G., Horrocks V., Miguéns Blanco J., Marchesi J.R., Mullish B.H., Clarke T.B., McDonald J.A.K. (2025). Vancomycin-Resistant Enterococci Utilise Antibiotic-Enriched Nutrients for Intestinal Colonisation. Nat. Commun..

[177] Cole C.G., Zhang Z.J., Dommaraju S.R., Dong Q., Pope R.L., Son S.S., McSpadden E.J., Woodson C.K., Lin H., Dylla N.P. (2025). Lantibiotic-Producing Bacteria Impact Microbiome Resilience and Colonization Resistance. Cell Host Microbe.

[178] Hausman B.S., Kaple C.E., Cadnum J.L., Memic S., Sangwan N., Donskey C.J. (2026). Longer Durations of Piperacillin/Tazobactam Treatment Cause More Prolonged Alteration of Colonization Resistance in Mice. PLoS ONE.

[179] Grießhammer A., de la Cuesta-Zuluaga J., Müller P., Gekeler C., Homolak J., Chang H., Schmitt K., Planker C., Schmidtchen V., Gallage S. (2025). Non-Antibiotics Disrupt Colonization Resistance against Enteropathogens. Nature.

[180] Bublitz A., Brauer M., Wagner S., Hofer W., Müsken M., Deschner F., Lesker T.R., Neumann-Schaal M., Paul L.S., Nübel U. (2023). The Natural Product Chlorotonil a Preserves Colonization Resistance and Prevents Relapsing *Clostridioides difficile* Infection. Cell Host Microbe.

